# The Vestibular System Implements a Linear–Nonlinear Transformation In Order to Encode Self-Motion

**DOI:** 10.1371/journal.pbio.1001365

**Published:** 2012-07-24

**Authors:** Corentin Massot, Adam D. Schneider, Maurice J. Chacron, Kathleen E. Cullen

**Affiliations:** 1 Department of Physiology, McGill University, Montreal, Quebec, Canada; 2 Department of Physics, McGill University, Montreal, Quebec, Canada; 3 Center for Applied Mathematics in Biology and Medicine, McGill University, Montreal, Quebec, Canada; University of Minnesota, United States of America

## Abstract

Although it is well established that the neural code representing the world changes at each stage of a sensory pathway, the transformations that mediate these changes are not well understood. Here we show that self-motion (i.e. vestibular) sensory information encoded by VIIIth nerve afferents is integrated nonlinearly by post-synaptic central vestibular neurons. This response nonlinearity was characterized by a strong (∼50%) attenuation in neuronal sensitivity to low frequency stimuli when presented concurrently with high frequency stimuli. Using computational methods, we further demonstrate that a static boosting nonlinearity in the input-output relationship of central vestibular neurons accounts for this unexpected result. Specifically, when low and high frequency stimuli are presented concurrently, this boosting nonlinearity causes an intensity-dependent bias in the output firing rate, thereby attenuating neuronal sensitivities. We suggest that nonlinear integration of afferent input extends the coding range of central vestibular neurons and enables them to better extract the high frequency features of self-motion when embedded with low frequency motion during natural movements. These findings challenge the traditional notion that the vestibular system uses a linear rate code to transmit information and have important consequences for understanding how the representation of sensory information changes across sensory pathways.

## Abstract 2

Early vestibular processing in macaque monkeys is inherently nonlinear and is optimized to detect specific features of self-motion.

## Author Summary

Understanding how the coding of sensory information changes at different stages of sensory processing remains a fundamental challenge in systems neuroscience. Here we address this question by studying early sensory processing in vestibular pathways of monkeys, a system for which sensory stimuli are relatively easy to describe. Peripheral vestibular afferents detect and encode head motion in space to ensure posture and gaze is accurate and stable during everyday life. In this study, we show that central vestibular neurons nonlinearly integrate their afferent inputs, which helps explain the mechanisms that generate enhanced feature detection in sensory pathways. In addition, our results overturn conventional wisdom that early vestibular processing is linear, revealing a striking boosting nonlinearity that is a hallmark of the first central stage of vestibular processing. Studies from other sensory systems have shown that higher-order neurons can more efficiently detect specific features of sensory input, and that nonlinear transformations can increase this efficiency. We suggest that nonlinear integration of afferent input by central vestibular neurons extends their coding range and facilitates the detection of natural vestibular stimuli.

## Introduction

Multiple representations of the sensory environment are found across the hierarchical stages of sensory systems [1]. Each of these representations is defined by the activities of a population of neurons in response to their afferent inputs. How neurons decode and then encode sensory information, and the ways in which neural strategies for coding change across successive brain areas, remains a central problem in neuroscience. Studies across sensory systems have shown that representations in higher order brain areas are more efficient because individual neurons detect specific features of sensory input [2]–[5]. Although theoretical studies predict that more efficient representations are achieved by nonlinear transformations of afferent input [3],[6],[7], to date the nature of these transformations is largely unknown.

If nonlinear transformations mediate a more efficient representation of the sensory environment across hierarchical stages of processing, then they should be revealed by experimental approaches specifically designed to probe nonlinear processing. Here, we used the vestibular system as a model to address whether central neurons nonlinearly integrate their afferent inputs in order to give rise to enhanced feature detection. An advantage of the vestibular system, which is essential for providing information about our self-motion and spatial orientation relative to the world, is that the sensory stimulus is relatively easy to describe. Conventional wisdom is that early vestibular processing is inherently linear. This is supported by numerous studies showing that both afferents and central neurons accurately encode the detailed time course of horizontal rotational head motion through linear changes in firing rate over a wide range of frequencies (reviewed in [8],[9]; [10]). Further support for this proposal has come from the fact that central vestibular neurons linearly transduce synaptic inputs into changes in firing rate output [11]. Indeed, to date, prior studies have demonstrated remarkable linearity of vestibular behaviours such as the vestibulo-ocular reflex [12]–[15]. However, all these results are at odds with the expectation that central vestibular neurons achieve more efficient representations of sensory space through nonlinear transformations of their afferent input. Such nonlinear transformations could be advantageous as they would enable vestibular neurons to detect specific features of natural vestibular stimuli. For instance, it would be theoretically beneficial that the central vestibular neurons which mediate vestibulo-spinal reflexes preferentially respond to unexpected transient stimuli, such as those experienced when slipping on ice, in order to optimize compensatory postural responses.

A comprehensive rethinking of the neural code used by the vestibular system is thus necessary to reveal whether more efficient representations of the sensory environment emerge in central vestibular pathways through nonlinear transformations of their afferent input. Notably, prior experiments have characterized early vestibular processing mostly using stimuli that were not designed to systematically probe nonlinear behaviour (e.g., single sinewaves and trapezoids) [8]–[10]. In order to test for the existence of such nonlinear transformations, it is necessary to compare neural response to a given stimulus “A” when presented in isolation to that obtained when the same stimulus was presented concurrently with another stimulus “B.” If, as suggested by previous studies, central vestibular neurons respond linearly, then we would expect that the response to stimulus “A” should not depend on whether stimulus “B” is present or not (i.e., the principle of superposition is valid because, by definition, a linear system must be additive). If, instead, central vestibular neurons nonlinearly integrate afferent input, we might expect that the response to stimulus “A” would be altered contingent on the presence of stimulus “B.”

We explicitly investigated how the neural strategy for coding self-motion changes across the afferent-central neuron synapses by testing whether central vestibular neurons nonlinearly integrate their afferent inputs. We found that, unlike afferents, central vestibular neurons do not obey the principle of superposition because they displayed strong nonlinear responses when sums of low and high frequency stimuli were used. Indeed, the response to low frequency stimuli was strongly attenuated when these were presented concurrently with high frequency stimuli. Through a combination of mathematical modeling and analysis, we show how a static boosting nonlinearity in the input-output relationship can lead to this effect. Our results force a rethinking of the processing of self-motion stimuli in early vestibular pathways. We suggest that nonlinear processing by central vestibular neurons could serve to enhance their coding range and selectivity to high frequency transient self-motion.

## Results

### Central Vestibular Neurons Respond Nonlinearly to Self-Motion

**Figure 1:**
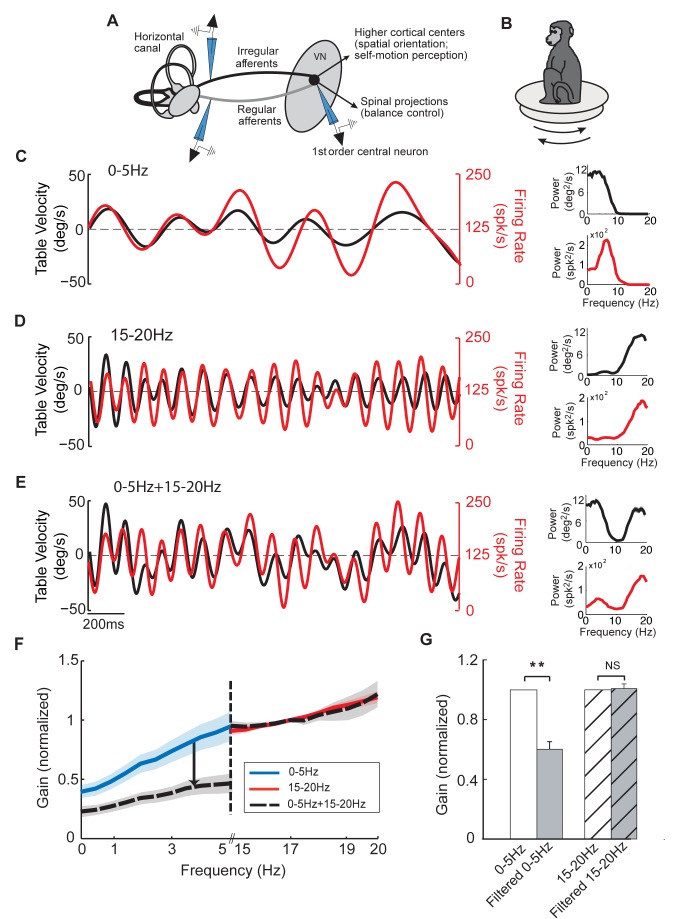
Central vestibular neurons respond nonlinearly to sums of noise stimuli. (A) Vestibular information is transmitted from the sensory end organs through two types of afferents (regular and irregular) that converge on first order central neurons within the vestibular nuclei. (B) During the experiment the monkey was comfortably seated in a chair placed on a motion platform. (C–E) The firing rate (red traces) of an example central vestibular neuron in response to noise stimuli (black traces) whose frequency content spanned 0–5 Hz (C), 15–20 Hz (D), and 0–5 Hz+15–20 Hz (E). The upper insets show the power spectrum of each stimulus, while the lower insets show the power spectrum of the firing rates (red). (F) Population-averaged normalized gains curves for central neurons. Note the attenuated response at low frequency (0–5 Hz, arrow). (G) Population-averaged normalized gains for central neurons. Here and in all subsequent figures, the bands (F) and error bars (G) show 1 SEM. The firing rate estimates were obtained by convolving the spike trains with a Kaiser filter (see Materials and Methods).

We tested response nonlinearity in both central vestibular neurons and afferents by recording their activities in response to a stimulus when presented in isolation and when presented concurrently with another stimulus (Figure 1A). During experiments, the animal was comfortably seated on a motion platform (Figure 1B). We first recorded central vestibular neuron responses to random noise stimuli with frequency content spanning the range of natural head rotations (0–20 Hz) [14]. Specifically, we applied stimuli that spanned two different frequency ranges: low (0–5 Hz) (Figure 1C, black traces) and high (15–20 Hz) (Figure 1D, black traces). Both noise stimuli were applied either individually (Figure 1C,D) or simultaneously (Figure 1E). The neuronal responses from an example cell to each of these three stimuli are shown by the red traces in Figure 1C,D,E. We found that, when both stimuli were applied simultaneously, the response was not equal to the sum of the responses to each individual stimulus as would be expected for a linear system. This is because the firing rate modulation in response to the low frequency stimulus when presented alone was much larger than that observed when the high frequency stimulus was presented simultaneously (compare red traces in Figure 1C,E). In contrast, the firing rate modulation in response to the high frequency stimulus was comparable regardless of whether the stimulus was presented alone or in combination with the low frequency input (compare red traces in Figure 1D,E). This was reflected in the response power spectrum (compare red traces in the insets of Figure 1C,E and Figure 1D,E).

To quantify this effect, we computed the response gain in each condition for our population of central vestibular neurons (see Materials and Methods). Consistent with previous results [10], the neuronal gains of central vestibular neurons were higher for high frequency stimuli (Figure 1F, compare blue and red traces). However, we found that the population-averaged response gains at low frequencies were significantly attenuated (∼50%) (*p*<10^−6^, paired *t* test, *n* = 15) when both stimuli are applied simultaneously (Figure 1F,G). The population-averaged response gains at high frequencies were, however, unaffected (*p* = 0.4, paired *t* test, *n* = 15) (Figure 1F,G).

Thus, contrary to the common assumption that early vestibular processing is essentially linear, the results above establish that central vestibular neurons respond nonlinearly to sums of low and high frequency head rotations since the principle of superposition is violated. Notably, responses to low frequency self-motion are suppressed in the presence of high frequency self-motion. In contrast, responses to high frequency self-motion are relatively unaffected by the presence of low frequency self-motion.

We next asked whether the response nonlinearity that we observed using gain measures would also be evident when using information theoretic measures such as the coherence. Unlike gain measures, coherence measures are computed using the signal-to-noise ratio and thus take variability into account. This is important because previous studies have shown that a given neuron can display qualitatively different frequency tuning depending on whether gain or coherence measures are used [16]–[18]. Again, we found that the principle of superposition was violated. Indeed, population-averaged coherence values at low frequencies were significantly lower (∼50%) (*p*<0.001, paired *t* test, *n* = 20) when both noise stimuli were presented simultaneously. In contrast, population-averaged coherence values at high (15–20 Hz) frequencies were not significantly different (*p* = 0.87, paired *t* test, *n* = 15) (Figure S1A, S1B, S1C). As expected given that there is a one-to-one relationship between coherence and mutual information measures, comparable results were obtained when computing the latter (unpublished data). Thus, taken together, our results using both gain and coherence measures confirm our hypothesis that central vestibular neurons respond nonlinearly to sums of low and high frequency stimuli.

We also tested that these nonlinear responses were not specific to the noise stimuli used. Indeed, we found that central vestibular neurons also responded nonlinearly to sums of low and high frequency sinusoidal stimuli. Indeed, when 3 and 17 Hz sinusoidal stimuli were applied simultaneously, the response was not equal to the linear sum of the responses to each individual stimulus (Figure S2). We note that this is not due to our filtering the spike trains to obtain the time-dependent firing rate since this effect was also evident in the power spectra from the unfiltered spike trains (Figure S3).

Further, the observed nonlinear responses of central vestibular neurons were not due to trivial nonlinearities such as rectification (i.e., cessation of firing) or saturation (i.e., the firing rate reaching a plateau at a finite value) since these were not elicited by the stimuli used in this study (Figure S4A).

### Peripheral Vestibular Afferents Respond Linearly to Sums of Low and High Frequency Motion

Perhaps the simplest explanation for the nonlinear responses of central vestibular neurons shown in Figure 1 is that they are inherited from their afferent input. Peripheral vestibular afferents display marked heterogeneities in their baseline activity and response to stimulation. Most notably, regularly discharging afferents are characterized by low coefficients of variation (CV) and encode the detailed time course of self-motion as they are broadly tuned to the behaviourally relevant frequency range (0–20 Hz). In contrast, irregularly discharging afferents are characterized by higher CVs and detect fast transient changes in self-motion as they respond preferentially to high frequencies [8],[18]–[20].

**Figure 2:**
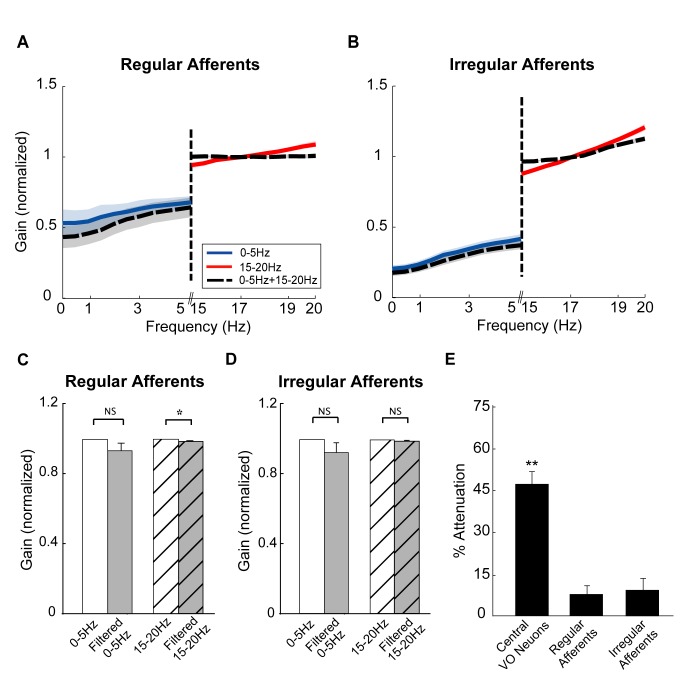
Afferents respond linearly to sums of noise stimuli. (A, B) Population-averaged normalized gain curves as a function of frequency for regular (A) and irregular (B) afferents. (C, D) Population-averaged normalized gains for regular (C) and irregular (D) afferents. (E) Population-averaged attenuation indices for central neurons, regular afferents, and irregular afferents.

To address whether the nonlinear responses of central vestibular neurons are inherited from their afferent inputs, we recorded from single regular and irregular afferents using the same random noise stimuli. In contrast to their target central vestibular neurons, neither regular (Figure 2A) nor irregular afferents (Figure 2A) displayed significant nonlinearities. Indeed, the population-averaged gain values at low frequencies were not significantly altered by the presence of the high frequency stimulus (regular: *p* = 0.9, paired *t* test, *n* = 5; Figure 2C; irregular: *p* = 0.23, paired *t* test, *n* = 10; Figure 2D). Similarly, the population-averaged gain values at high frequencies were not significantly altered by the presence of the low frequency stimulus (regular: *p* = 0.84, paired *t* test, *n* = 5; irregular: *p* = 0.19, paired *t* test, *n* = 10). We note that the applied stimuli also did not elicit “trivial” nonlinearities in afferents such as rectification or saturation (Figure S4B,C) and that similar results were obtained when we instead used the coherence measure (regular: Figure S1D,E,F; irregular: Figure S1G,H,I). We note that similar results were observed when using sums of low and high frequency sinusoidal stimuli (unpublished data). Accordingly, unlike central neurons, individual afferents do not respond nonlinearly to sums of low and high frequency stimuli.

We quantified the gain attenuation at low frequencies in the presence of the high frequency stimulus for both central vestibular neurons and afferents. While central vestibular neurons displayed strong and significant attenuation (∼50%, *p*<0.001, signrank test, *n* = 15), both regular and irregular afferents instead displayed weak attenuation (∼10%) that was not significantly different from zero (regular: *p* = 0.25, signrank test, *n* = 5; irregular: *p* = 0.13, signrank test, *n* = 10) (Figure 2E). These findings imply that the origin of the response nonlinearity seen in central neurons is due to nonlinear integration of afferent synaptic input.

### Central Vestibular Neurons Display Nonlinear Responses to High Frequency But Not Low Frequency Head Rotations When These Are Applied in Isolation

**Figure 3:**
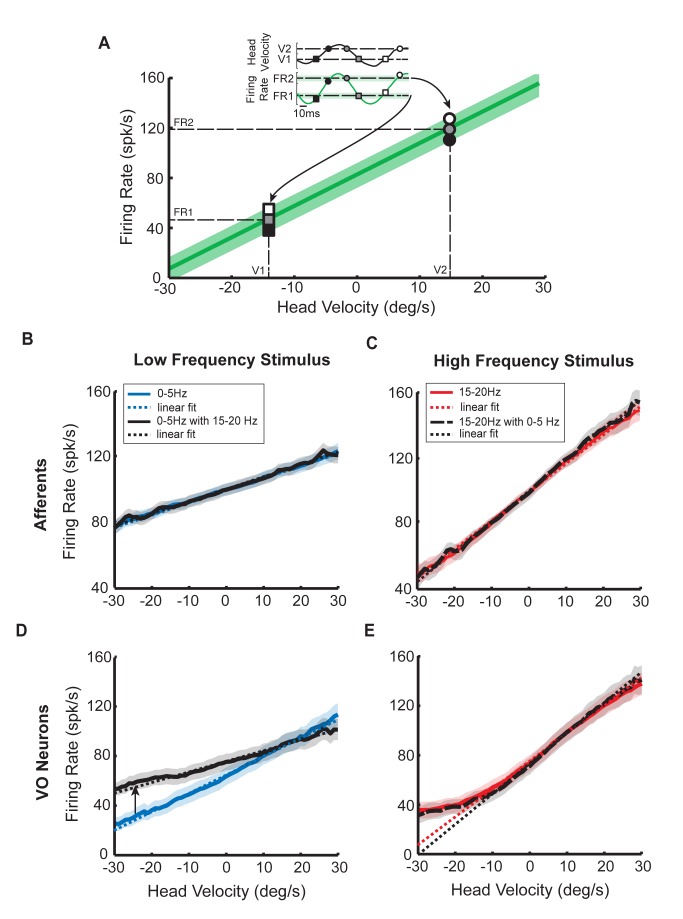
Central vestibular neurons but not afferents display a nonlinear relationship between output firing rate and input head velocity. (A) Output firing rate as a function of head velocity. The inset shows the instantaneous firing rate and the head velocity stimulus as a function of time and the various symbols correspond to different values of the head velocity and the corresponding firing rates. If the firing rate is related linearly to the head velocity stimulus, then the curve relating the two should be well fit by a straight line. The slope of this line is then the response gain. (B) Population-averaged firing rate response as a function of head velocity for afferents when stimulated with 0–5 Hz noise alone (solid blue) and concurrently with 15–20 Hz noise (solid black). In both cases, the curves were well fit by straight lines (dashed lines) and largely overlapped (0–5 Hz alone: *R* ^2^ = 0.99, slope = 0.70 (spk/s)/(deg/s), *y*-intercept = 98 spk/s; 0–5 Hz with 15–20 Hz: *R* ^2^ = 0.99, slope = 0.72 (spk/s)/(deg/s), *y*-intercept = 98 spk/s). (C) Population-averaged firing rate response as a function of head velocity for afferents when stimulated with 15–20 Hz noise alone (solid red) and concurrently with 0–5 Hz noise (long dashed black). Both curves were again well fit by straight lines (short dashed lines) and largely overlapped (15–20 Hz alone: *R* ^2^ = 0.99, slope = 1.97 (spk/s)/(deg/s), *y*-intercept = 102 spk/s; 15–20 Hz with 0–5 Hz: *R* ^2^ = 0.99, slope = 2.06 (spk/s)/(deg/s), *y*-intercept = 102 spk/s). Note, however, the increased slope with respect to panel B. (D) Population-averaged firing rate response as a function of head velocity for central neurons when stimulated with 0–5 Hz noise alone (solid blue) and concurrently with 15–20 Hz noise (solid black). In both cases, the curves were well fit by straight lines (dashed lines) although the solid black curve had a lower slope (i.e., gain) than the solid blue curve (0–5 Hz: *R* ^2^ = 0.98, slope = 1.56 (spk/s)/(deg/s), *y*-intercept = 67 spk/s; 0–5 Hz with 15–20 Hz: *R* ^2^ = 0.87, slope = 0.83 (spk/s)/(deg/s), *y*-intercept = 81 spk/s). (E) Population-averaged firing rate response as a function of head velocity for central neurons when stimulated with 15–20 Hz noise alone (solid red) and concurrently with 0–5 Hz noise (long dashed black). While both curves were similar and largely overlapped, they were not well fit by straight lines (short dashed lines) that underestimated the firing rate for head velocities <−10 deg/s (15–20 Hz: *R* ^2^ = 0.64, slope = 2.32 (spk/s)/(deg/s), *y*-intercept = 79 spk/s; 15–20 Hz with 0–5 Hz: *R* ^2^ = 0.27, slope = 2.78 (spk/s)/(deg/s), *y*-intercept = 79 spk/s). We note that central neurons did not display rectification since the firing rate was always above zero.

In order to understand how central vestibular neurons nonlinearly integrate their afferent input, we next characterized the relationship between head velocity input and output firing rate for both afferents and central neurons by plotting one as a function of the other. The schematic of the approach used is illustrated in Figure 3A. If the relationship between input head velocity and output firing rate is linear, then the curve relating the two should be well fit by a straight line.

We found that the relationships between head velocity stimuli and peripheral afferent responses were well fit by straight lines. The population-averaged relationships for low and high frequency self-motion obtained for afferents are shown in Figure 3B and 3C, respectively. It can further be seen that these relationships are comparable when a given stimulus is applied alone and when it is applied concurrently with the other stimulus (Figure 3B, 3C) (low frequency: *p* = 0.93, pairwise *t* test, *n* = 15; high-frequency: *p* = 0.89, pairwise *t* test, *n* = 15), demonstrating that the principle of superposition applies. This was also seen for single neurons (insets of Figure S5). Further, these results were observed for both regular (low frequency: *p* = 0.59; high frequency: *p* = 0.58, pairwise *t* tests, *n* = 5) and irregular (low frequency: *p* = 0.77; high frequency: *p* = 0.35, pairwise *t* tests, *n* = 10) afferents when considered separately (Figure S5). Notably, comparison of Figure 3B and 3C further revealed that the afferent gain (i.e., the slope of the input-output relationship) was higher in response to the high as compared to the low frequency stimulus. This observation is consistent with previous studies showing that high frequency head rotations give rise to greater afferent firing rate modulations (reviewed in [8]).

We next computed the population-averaged relationships for central vestibular neurons and found that they were well fit by straight lines when the low frequency stimulus was presented alone (Figure 3D, solid blue curves). We note that this was also true for single neurons (Figure S6A, solid blue curve). The head velocity-neuronal response relationship (solid black curve) was also linear when low frequency stimulation was applied concurrently with high frequency stimulation (population average: Figure 3D; single neuron: Figure S6A, solid black curves). However, in the combined condition, the slope of the curve (i.e., the gain) was lower (compare solid black and blue traces in Figures 3D and S6A). These results are consistent with our previous analysis of response gain (Figure 1G), thus confirming our earlier findings.

In contrast, qualitatively different results were observed for high frequency head rotations. Notably, we found that the relationships between head velocity stimuli and central neuron responses were nonlinear as they were characterized by significantly lower gains (i.e., the slope of the curve) for head velocities less than −10 deg/s as compared to those for head velocities greater than −10 deg/s (*p* = 0.01, pairwise *t* test, *n* = 20). This was seen for both the population averages (Figure 3E) and single neurons (Figure S6B). We will henceforth refer to the shape of these curves as a boosting nonlinearity [21]. Moreover, the relationships obtained for high frequency head rotations were comparable when the stimulus was presented alone or concurrently with low frequency head rotations (*p* = 0.43, pairwise *t* test, *n* = 20) (Figures 3E and S6B, compare red and black-dashed traces).

Thus, again consistent with our results using gain measures, central vestibular neuron responses were comparable when high frequency stimuli were applied alone or concurrently with low frequency stimuli. Notably, unlike afferents, central vestibular neurons respond nonlinearly to sums of low and high frequency stimuli. Moreover, our analysis of their stimulus input–firing rate output relationships further revealed a boosting nonlinearity characterized by lower slopes for head velocities less than −10 deg/s as compared to those obtained for head velocities greater than −10 deg/s. This nonlinearity was only seen when high frequency stimuli were applied (Figures 3E and S6B).

### The Greater Afferent Firing Rate Modulations Elicited by High Frequency Stimuli Elicit Nonlinear Responses in Central Vestibular Neurons

**Figure 4:**
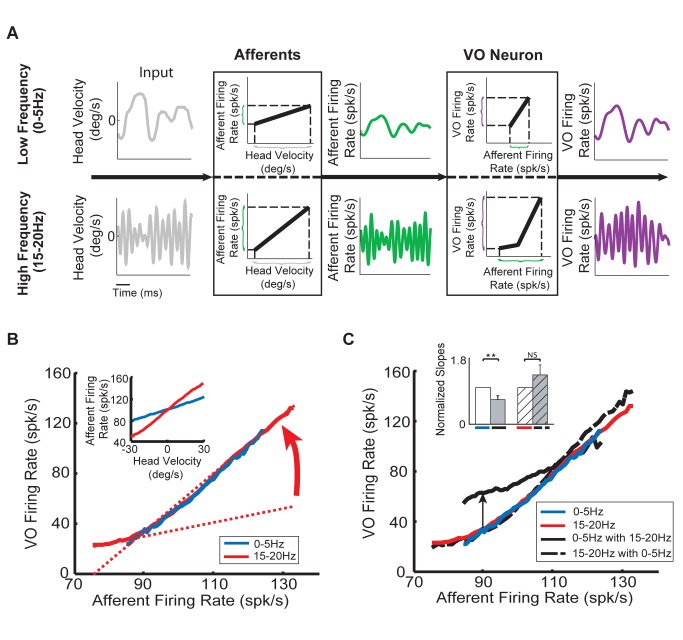
Central neurons display a static nonlinear relationship between their output firing rate and their afferent input. (A) Low (top) and high (bottom) frequency head velocity stimuli (gray) cause smaller and larger changes in afferent firing rate (green), respectively. These differential changes in afferent firing rate in turn cause differential changes in central neuron firing rate (purple), respectively. Notably, the changes in afferent firing rate caused by high frequency head velocity stimuli are distributed over a greater range and thus elicit nonlinear responses from VO neurons, whereas this is not the case for those caused by low frequency head velocity stimuli. Note that the same scales were used for corresponding panels in the bottom and upper rows. (B) Population-averaged firing rates of central VO neurons as a function of afferent firing rate for low (blue) and high (red) frequency noise stimuli presented in isolation. Note that the curve obtained for the low frequency stimulus (blue) extends over a smaller range than that obtained for high frequency (red) stimuli. Further, both curves are linear over the range for which they overlap. Also shown are best linear fits to the portion of the curve below and above 90 Hz (dashed red lines). As such, the curve can be approximated by a piecewise linear function. Inset: population-averaged firing rates of afferents as a function of the head velocity stimulus for low (blue) and high (red) frequency noise stimuli presented alone. (C) Population-averaged firing rates of central VO neurons as a function of afferent input firing rates: (1) for the low frequency stimulus when presented alone (blue) and concurrently with the high frequency stimulus (solid black); (2) for the high frequency stimulus when presented alone (red) and concurrently with the low frequency stimulus (dashed black). Note that the curves obtained in response to the high frequency stimulus when presented alone (red) and when presented concurrently with the low frequency stimulus (dashed black) overlapped before (Figure 3E) and thus, not surprisingly, also overlap. Note also that only the curve obtained when the low frequency stimulus was presented concurrently with the high frequency stimulus (solid black) does not overlap with the others. This is because the central VO neuron firing rate is higher than that obtained for the low frequency stimulus when applied alone for values lesser than 110 Hz. Inset: population-averaged normalized slopes under all four conditions. The afferent activity was estimated by fitting a linear model to previous experimental recordings from a large population of afferents (see Materials and Methods).

Thus far, we have looked at the relationship between head velocity stimuli and output firing rates for both central neurons and afferents. We found that afferents responded linearly to both low and high frequency stimuli. In contrast, central neurons responded linearly to low frequency stimuli but nonlinearly to high frequency stimuli. A priori, this effect could be mediated by a dynamic non-linearity that would be activated exclusively under high frequency stimulation (e.g., a network-based mechanism such as feedback input from higher centers). Alternatively, the nonlinearity might be static in nature (e.g., due to intrinsic mechanisms such as voltage-gated conductances) and be preferentially elicited by the afferent input due to high frequency stimulation. Figure 4A illustrates the sequential processing of low (top) and high (bottom) frequency stimuli when applied in isolation. It is important to note that, for high frequency stimulation, the afferent input to central vestibular neurons will span a greater range (Figure 4A, compare green traces) because afferents display greater sensitivities (compare Figure 3B,C). As a result, at the next stage of processing, these larger afferent firing rate modulations should evoke greater central neuron firing rate modulations as compared to those evoked by low frequency head rotations (Figure 4A, compare purple traces). Thus, if the nonlinearity is static, we predict that (1) the smaller range of afferent firing rates evoked by low frequency stimulation are contained in a region for which the central vestibular neuron input-output relationship is approximately linear, (2) the greater range of afferent firing rates evoked by high frequency stimulation extend into a region of the input-output relationship that elicits the boosting nonlinearity (Figure 4A, VO neuron box), and as a result, (3) central vestibular neuron output firing rate is then a fixed function of the afferent input firing rate, regardless of whether low or high frequency head rotations are applied in isolation.

To test whether the nonlinearity is static or dynamic, we next experimentally characterized the input-output relationship of central neurons by plotting their output firing rates as a function of their afferent input rather than head velocity. Given that central neurons receive input from many afferents that display significant heterogeneities (see [8] for review), we obtained an estimate of this activity by fitting a linear model to previous data (see Materials and Methods). The input-output relationship obtained for low frequency stimuli was approximately linear (Figure 4B, blue curve), confirming our first prediction. In addition, the input-output relationship obtained for high frequency stimuli displayed a boosting nonlinearity (Figure 4B, red curve), such that the slope for afferent inputs less than 90 spk/s was much lower than that for afferent inputs greater than 90 spk/s (Figure 4B, compare solid and dashed red curves). Thus, the afferent input–central neuron output relationship can be approximated by the piecewise linear function illustrated in Figure 4A, confirming our second prediction. Moreover, we found that both curves overlapped when only the smaller range of afferent firing rates evoked by low frequency stimuli was considered (Figure 4B, compare red and blue curves). Accordingly, this finding confirmed our third prediction that central vestibular neuron firing rate is a fixed function of the afferent input firing rate when either low or high frequency head rotations are applied in isolation. Accordingly, there is a striking contrast between the results of this analysis and that of our previous analysis of the relationship between head velocity input and afferent output. Notably, the head velocity input–afferent output relationships obtained for low and high frequency stimulation did not overlap consistently with the known frequency-dependent sensitivities of afferents (Figure 4B, inset). Thus, taken together, our results show that central vestibular neuron responses are characterized by a static nonlinearity that is primarily elicited by the greater afferent firing rate modulations caused by high frequency stimuli. We suggest that the intrinsic properties of central vestibular neurons and/or network interactions within this vestibular pathway underlie this boosting nonlinearity (see Discussion).

We next plotted the afferent input–firing rate output relationships obtained when low frequency stimulation was applied alone or concurrently with high frequency stimulation for central vestibular neurons. We found significantly different slopes in both conditions (Figure 4C, compare black and blue curves and inset). Specifically, central vestibular neuron firing rates in response to afferent firing rates below 110 spk/s were higher when the low frequency stimulus was applied concurrently with the high frequency stimulus than when it was applied alone (Figure 4C, arrow). We also note that, as can be expected from Figure 3E, the central vestibular neuron input-output relationships obtained when high frequency stimulation was applied alone or concurrently with low frequency stimulation overlapped (Figure 4C, red and dashed black curves) and did not differ significantly in their slopes (Figure 4C, inset), which confirms that central vestibular neurons display a static boosting nonlinearity in response to these stimuli.

### Modeling and Predicting Central Vestibular Neuron Responses to Sums of Arbitrary Stimuli

**Figure 5:**
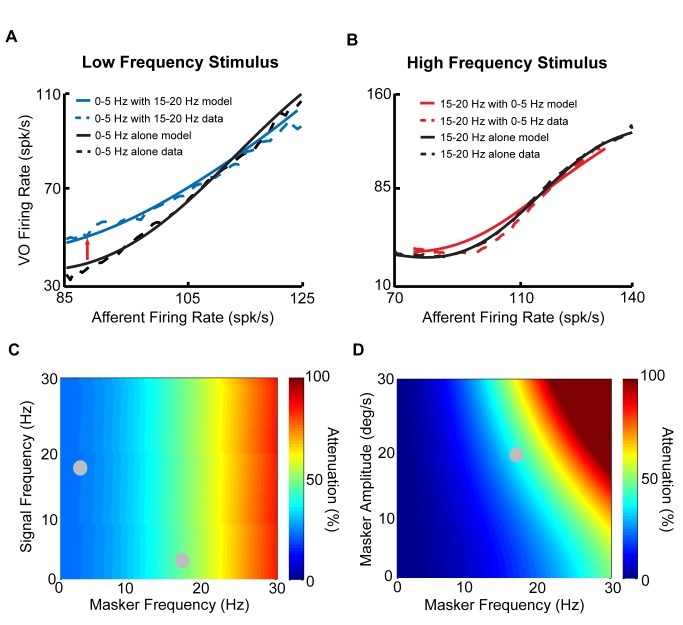
A simple model accurately predicts nonlinear central VO neuron responses to sums of low and high frequency stimuli. (A) Model (solid) and data (dashed) relationships between afferent firing rate and central VO neuron firing rate when the low frequency stimulus was presented alone (blue) and concurrently with the high frequency stimulus (black). Note that the model accurately reproduces the decrease in slope seen experimentally as evidenced by the large overlap between the model and data curves (*R* ^2^ = 0.92). (B) Model (solid) and data (dashed) relationships between afferent firing rate and VO neuronal firing rate when the high frequency stimulus was presented alone (red) and concurrently with the low frequency stimulus (black). Note that the model also accurately reproduces the lack of change seen experimentally as the model curves largely overlap with the experimental ones (*R* ^2^ = 0.99). (C) % gain attenuation plotted as a function of signal and masker frequency. The stimulus for which the response is computed is referred to as the signal, while the other stimulus is referred to as the masker. Maskers with higher frequency content lead to greater gain attenuation. (D) % gain attenuation as a function of masker amplitude and frequency. Maskers of greater amplitude and frequency lead to greater gain attenuation.

Does the static boosting nonlinearity in the input-output relationship of central vestibular neurons account for their nonlinear responses to sums of low and high frequency stimuli? To address this question, we fit the experimentally recorded central vestibular neuron input-output relationship in response to afferent input when a given stimulus was presented in isolation. Since individual central vestibular neurons receive input from a large heterogeneous population of afferents [8], we estimated their average activity by fitting a linear model to existing data (see Materials and Methods). The input-output relationship in response to this stimulus when another stimulus is presented concurrently can then be obtained by averaging (see Materials and Methods). Accordingly, it becomes possible, using this model, to predict the change in the central vestibular neuron input-output relationship to a given stimulus when another stimulus is applied concurrently. Our results show that, when compared to experimental data, this relatively simple model is surprisingly accurate at predicting the change in afferent to central neuron input-output relationship to the low frequency stimulus when the high frequency stimulus is applied concurrently (Figure 5A, compare solid and dashed curves). The same model also predicts little change in the input-output relationship to the high frequency stimulus when the low frequency stimulus is applied concurrently, consistent with our experimental results (Figure 5B, compare solid and dashed curves).

Importantly, using this model, we were further able to predict the relative gain attenuation in response to sums of stimuli with given intensities and frequencies within the behaviourally relevant range. It then becomes important to introduce new terminology to distinguish both stimuli by other means than just their frequency content, as was done until now. Thus, we will henceforth refer to one stimulus as the “signal” and to the other as the “masker.” Note that, while the terms “signal” versus “masker” are arbitrary, this division allows us to focus on the coding of one input (i.e., the input designated as the signal). Our model shows stronger attenuation of the response gain to a low frequency signal by maskers with higher frequency content (Figure 5C). This is because vestibular afferents display gains that increase as a function of frequency. Moreover, our model shows stronger attenuation of the response gain to a given signal by maskers with higher intensity (Figure 5D). This is because maskers of greater intensities are more effective at eliciting nonlinear responses from central vestibular neurons. Thus, although it is not experimentally feasible to test all combinations of maskers and signals, our model allows us to make testable predictions of how a static nonlinear input-output relationship attenuates central vestibular neuron responses to a given signal in the presence of a masker over the physiologically relevant range of frequencies and intensities. For example, our model makes the prediction that a masker with a given frequency content is equally effective at attenuating the sensitivity to signals with either low or high frequency content (Figure 5C).

### A Linear-Nonlinear Cascade Model Verifies That Central Vestibular Neurons Display a Static Boosting Nonlinearity

**Figure 6:**
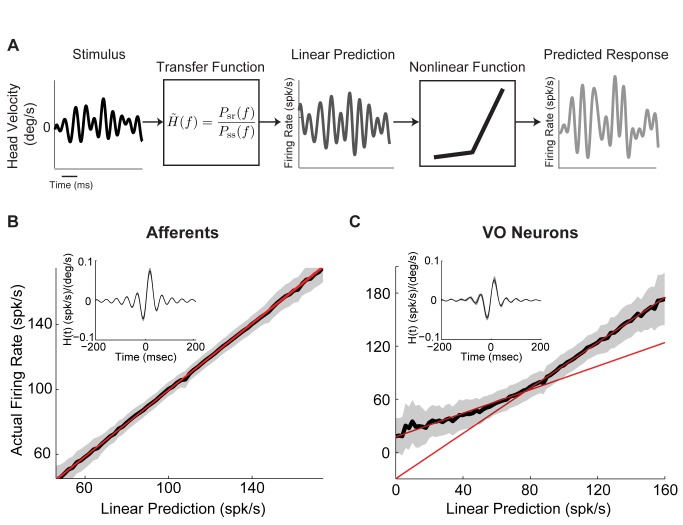
A linear-nonlinear (LN) cascade model reveals that central vestibular neurons respond nonlinearly to broadband noise stimulation. (A) Schematic showing the LN model's assumptions. The stimulus (left) is convolved with a filter *H(t)* that is given by the inverse Fourier transform of the transfer function 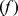 in order to generate the linear predicted firing rate (middle). This linear prediction is then passed through a static function f (which can be linear or nonlinear) to give rise to the predicted output firing rate (right). (B) Population-averaged function f for afferents. Also shown is the best-fit line (*R* ^2^ = 0.998±0.001, *n* = 15) (red) whose slope did not significantly differ from unity (*p* = 0.99, *n* = 15, pairwise *t* test). Inset: population-averaged filter *H(t)* for afferents. (C) Population-averaged function f for central VO neurons. Also shown are the best-fit straight lines for the intervals (0–80 Hz) and (80–160 Hz) (red) whose slopes were significantly different from one another (*p* = 0.0014, *n* = 13, pairwise *t* test). Inset: population-averaged filter *H(t)* for central VO neurons.

So far, our data and modeling results show that a static boosting nonlinearity can explain why central neurons display reduced gain to low frequency motion when applied concurrently with high frequency motion. If this is true, then central vestibular neurons should respond nonlinearly to any stimulus that contains high frequencies. Moreover, the form of nonlinearity should be stimulus independent. To test this prediction experimentally, we recorded from afferents and central vestibular neurons during broadband noise stimulation and used a more general approach to characterize their responses. Specifically, we used a linear-nonlinear (LN) cascade model [22] that is illustrated in Figure 6A (see Materials and Methods). This model assumes that a neuron's firing rate at any instant is a function f of the convolution between the stimulus and an optimal linear filter (i.e., the linear prediction) [22]. The form of the function f can then be estimated by plotting the actual firing rate as a function of the linear prediction (Figure 6A).

We first applied this model to our afferent data and found that their output firing rates were well predicted by the optimal linear filter alone as all data points were located close to the identity line (*R* ^2^ = 0.998±0.001, *n* = 15) (Figure 6B). This was seen for both regular (Figure S7A,B) and irregular (Figure S7C,D) afferents. Notably, the slope of best straight line fit to the curve (Figure 6B, red line) was not significantly different from unity (*p* = 0.966, *n* = 15, pairwise *t* test).

Qualitatively different results were obtained for central vestibular neurons. Indeed, we found that their output firing rates were not well predicted by the optimal linear filter alone (Figure 6C) as evidenced by significant deviations from the identity line (Figure S7E,F). Notably, the slope of the best straight line fit to the curve over the range (0–80 Hz) was significantly lower than the slope of the best straight line fit to the curve over the range (80–160 Hz) (*p* = 0.0014, *n* = 13, pairwise *t* test) (Figure 6C, compare red lines). Additionally, the curve relating the actual firing rate to the linear prediction in response to broadband noise stimuli closely resembled the nonlinear input-output relationship obtained in response to high frequency narrowband noise stimuli (compare Figures 6C and 3E), which suggests that the frequency filtering properties of central vestibular neurons are mostly inherited from afferents. The actual responses were well predicted by the full LN model (*R* ^2^ = 0.94±0.07, *n* = 13). We also note that the firing rate values extrapolated from the best straight line fit to the curve over the range (80–160 Hz) are negative over the range (0–20 Hz), while the actual firing rate values are of course positive. We shall return to this point in the discussion.

Finally, we compared the curves relating the actual firing rate to the linear prediction for afferents and central vestibular neurons for different stimuli (i.e., low frequency, high frequency, low+high frequency, and broadband noise stimuli). The afferent curves overlapped and were all located close to the identity line (Figure S8A), confirming that the responses were well fit by linear models. The curves for central vestibular neurons also overlapped, but exhibited significant deviations from linearity only for stimuli that contained high frequencies (Figure S8B). As such, our results using LN models provide additional strong evidence that central vestibular neurons indeed display a static boosting nonlinearity that is preferentially elicited by the greater afferent firing rate modulations caused by high frequency motion and that their frequency filtering properties are largely inherited from those of afferents.

### How Does a Static Boosting Nonlinearity Give Rise to Suppressed Response to Low Frequency Stimuli in the Presence of High Frequency Stimuli?

**Figure 7:**
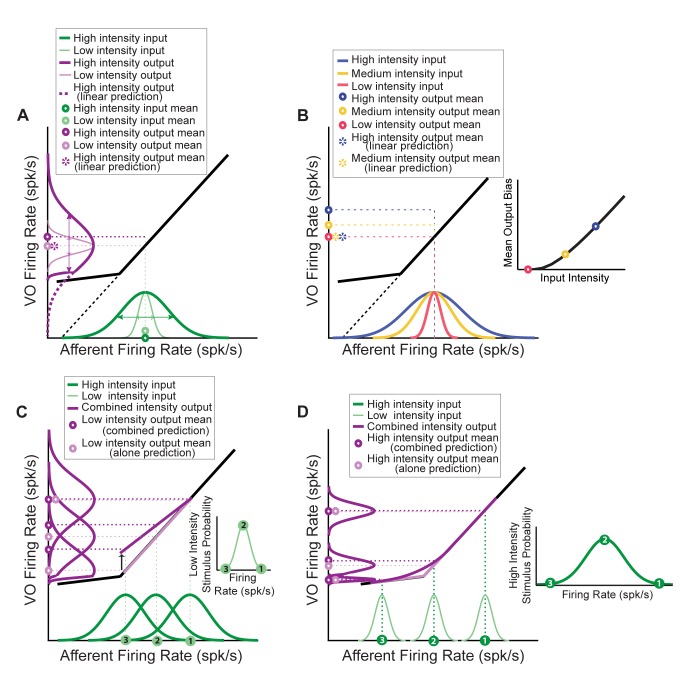
Schematic showing how a nonlinear static relationship between input and output can lead to attenuated sensitivity to sums of low and high frequency stimuli. (A) Input-output relationship showing a vertex (i.e., a sudden change in slope) (black curve). If we assume that the input is normally distributed with low intensity (i.e., standard deviation) such that all the input values are to the right of the vertex (light green distribution on *x*-axis), then the corresponding output distribution will also be normally distributed (light purple distribution on *y*-axis). The mean output (light purple circle on *y*-axis) corresponds to the image of the mean input (dashed purple circle on *y*-axis; note that the light purple and dashed purple circles were offset for clarity) as both input and output are linearly related. In contrast, for a higher intensity input that extends significantly past the vertex (dark green distribution on *x*-axis), the corresponding output distribution (dark purple on *y*-axis) is skewed with respect to the linear prediction (dashed purple on *y*-axis). The mean output (dark purple circle on *y*-axis) is thus greater than the linear prediction (dashed purple circle on *y*-axis). (Note that here and below, we represented the distributions to have the same maximum value in order to emphasize the fact that we are changing the standard deviation.) (B) Increasing the input distribution intensity for a given mean (compare red, yellow, and blue distributions) causes a greater skew in the corresponding output distribution (unpublished data) and thus an increased bias in their means (red, yellow, and blue dots on the *y*-axis and inset) as compared to the linear prediction (dashed yellow and blue dots on the *y*-axis). (C) Shifting the mean of the high intensity input distribution to the left (compare points 1, 2, and 3 on the *x*-axis and the inset) makes it extend to the left of the vertex more and more (compare the green curves on the *x*-axis), causing greater skewness in the corresponding output distributions (purple curves on the *y*-axis), which creates a greater bias in the mean (dark purple points on *y*-axis) with respect to the linear prediction (light purple points on *y*-axis). As a result, the mean output in response to a given value of the low intensity input (points 1, 2, and 3 on the *x*-axis) when the high intensity signal is present (dark purple line) has a lower slope (i.e., gain) than when the high intensity signal is absent (light purple line). (D) Shifting the mean of the high intensity input distribution to the left (compare points 1, 2, and 3 on the *x*-axis and the inset) makes the corresponding distributions of the low intensity input extend to the left of the vertex more and more (green curves on the *x*-axis), causing greater skewness in the output distribution (purple curves on the *y*-axis), which creates a greater bias in the mean (dark purple points on *y*-axis) with respect to the linear prediction (light purple points on *y*-axis). Note, however, that the bias in the mean will be lower than in (C) since the input distributions now have a lower intensity as explained in (B). Thus, the input-output relationship when the low intensity signal is present (dark purple line) will have a lower slope (i.e., gain) than when the low intensity signal is absent (light purple line) but the effect will be weaker than in (C).

Our results above have shown that a static boosting nonlinearity can indeed account for the nonlinear responses of central vestibular neurons. Here, we provide an intuitive explanation of how a static boosting nonlinearity leads to the experimentally observed response attenuation to low frequency stimuli when presented concurrently with high frequency stimuli. First, consider a piecewise linear input-output relationship between afferent firing rate and central neuron firing rate such as that illustrated in Figure 7A. If the afferent input is normally distributed with low intensity such that it is constrained to the right side of the vertex (i.e., the point at which the slope suddenly changes), then the corresponding output firing rate will be linearly related to the afferent input and thus will also be normally distributed (Figure 7A, distribution and mean plotted in light purple). This is the situation when low frequency stimuli are applied in isolation. In contrast, if a normally distributed afferent input has a greater intensity and thus spans a greater range of values extending past the vertex (e.g., when high frequency stimuli are applied), then the output firing rate will be a nonlinear function of the input and thus will not be normally distributed any longer. This is because the output firing rate distribution has become skewed, thus shifting its mean to higher values than what would be predicted if the input-output relationship were linear (Figure 7A, distribution and mean plotted in dark purple). Notably, the skew in the input-output distribution will increase as a function of the input distribution intensity (compare the three distributions in Figure 7B), which in turn will increase the bias in the mean with respect to what is expected if the distribution was linear (Figure 7B, inset). We note that, under experimental conditions, the input intensity will increase when the head velocity stimulus increases in either intensity or frequency content.

Why then does a skewed output distribution result in higher sensitivity to the low frequency stimulus when applied in isolation than when applied concurrently with the high frequency stimulus? To answer this question, note that the output firing rate in response to a given value of the afferent input firing rate caused by the low frequency stimulus must be averaged over the normal distribution of values of the high frequency stimulus. This is because both stimuli are not correlated. For a high value of the low frequency stimulus (point 1, Figure 7C), the distribution of the high frequency stimulus spans the linear range of the piecewise linear input-output relationship. As such, the average output firing rate in response to this value of the low frequency stimulus when presented concurrently with the high frequency stimulus is equal to that obtained when the low frequency stimulus is presented in isolation. However, this is not the case for lower values of the low frequency stimulus (points 2 and 3, Figure 7C). Indeed, in these cases, the distribution of the high frequency input extends past the vertex. As a consequence, the distribution of output firing rates is skewed as explained above. The average central vestibular neuron output in response to low values of the low frequency stimulus is thus greater than what would be expected if the input-output relationship were linear. Moreover, the skewness becomes greater for lower values of the low frequency stimulus (compare the purple output distributions corresponding to points 2 and 3, Figure 7C), resulting in a greater bias in the output firing rate. This bias, in turn, reduces the slope of the input-output relationship between output and input firing rates when the low frequency stimulus is presented concurrently with the high frequency stimulus, as compared to that obtained when the low frequency stimulus is presented in isolation.

Finally, the above argument leads to the crucial question of why central vestibular neurons display similar sensitivities to high frequency stimuli when applied in isolation or concurrently with low frequency stimuli. As illustrated in Figure 7D, low frequency stimuli will tend to give rise to narrower distributions of afferent input firing rates and thus smaller biases than high frequency stimuli because of the high-pass filtering characteristics of afferents (compare distributions in Figure 7D and 7C, respectively), thereby leading to smaller attenuations in sensitivity.

## Discussion

### Summary of Results

What is the neural code used by the brain to represent self-motion (i.e., vestibular) information? We showed that neurons at the first central stage of vestibular processing respond nonlinearly to sums of low and high frequency stimuli. This is because, when stimuli contained low and high frequency motion components, responses to the low frequency component were strongly attenuated. Given that such responses were not observed in afferents, we hypothesized that this occurs because central vestibular neurons nonlinearly integrate their afferent inputs. Computing input-output relationships revealed that afferent firing rates were related linearly to head velocity in all stimulation paradigms. In contrast, the relationship between head velocity and central neuron firing rate was characterized by a significant boosting nonlinearity for high frequency stimulation. Prior studies have shown that higher frequency stimuli elicit greater changes in afferent firing rate than do low frequency stimuli (reviewed in [8]). We hypothesized that this frequency-dependent afferent response plays a vital role in establishing the conditions for which central vestibular neurons will preferentially display nonlinear responses. We confirmed this hypothesis by plotting the central vestibular neuron firing rate output as a function of the afferent firing rate input, and then formulated a model to explain our findings. We then demonstrated the generality of this model by predicting neuronal responses to sums of arbitrary stimuli and conclude that high-pass filtering characteristics displayed by afferents combined with the nonlinear input-output relationship of central vestibular neurons underlie their attenuated responses to low frequency motion when presented concurrently with high frequency motion. To test that this boosting nonlinearity was indeed static and preferentially elicited by high frequency stimulation, we used LN cascade models to predict responses to broadband noise stimulation. We found that central vestibular neuron responses were well fitted by these models and that the form of the nonlinearity closely matched that obtained for high frequency narrowband noise stimulation with our previous analysis, suggesting that the frequency filtering properties of central vestibular neurons are mostly inherited from that of afferents. Finally, we provided an intuitive explanation as to why a static boosting nonlinearity can lead to the attenuation of the response to low frequency motion in the presence of high frequency motion. Specifically, the nonlinear response of central neurons to high frequency motion creates a skew in the output firing rate distribution, which increases its mean with respect to what would be expected if the input-output relationship was linear. This bias in turn decreases the input-output relationship slope when low frequency motion is presented concurrently with high frequency motion.

### Origins of the Nonlinear Processing in Early Vestibular Pathways

While our findings confirm that vestibular afferents display linear responses over a wide frequency range, they further show the novel result that central vestibular neurons respond nonlinearly to sums of low and high frequency stimuli, since they violate the principle of superposition. This is surprising given that previous reports have found that the high conductance state of neurons in vivo can have a significant influence on their processing of synaptic input through linearization in their input-output relations [23]–[26], which is thought to extend the neuronal coding range [27]. Our results further show that the nonlinear responses of central vestibular neurons to sums of low and high frequency self-motion are caused by a static boosting nonlinearity in their input-output relationships. This nonlinearity differs from those (directional asymmetry, soft saturation) described in prior studies examining the responses of these same neurons [28],[29]. We note that our stimuli were designed as to not elicit “trivial” nonlinearities such as rectification and saturation from both afferents and central vestibular neurons but that these will indeed be elicited by high intensity stimuli [30].

What causes the observed boosting nonlinearity in central vestibular neurons? Our results show that this nonlinearity is static, and thus support the hypothesis that it is caused by intrinsic mechanisms such as short-term synaptic plasticity [31], voltage-dependent conductances [32], or the diversity in the innervations patterns of regular versus irregular afferent inputs onto central vestibular neurons [33] rather than network mechanisms such as nonlinear inhibitory connections within the known recurrent feedback loops of the vestibular nuclei/cerebellum [34],[35]. It is, however, difficult to determine the exact nature of these mechanisms for several reasons. (1) Intrinsic mechanisms such as synaptic conductance, passive membrane properties, and voltage-gated currents of neurons in the vestibular nuclei have been primarily been studied in mouse and guinea pig (reviewed in [36]) and not in primates. This is important because previous studies have shown significant differences in the activities of rodent and monkey vestibular nuclei neurons in vivo [37]. (2) Most prior characterizations of intrinsic mechanisms were performed under in vitro conditions, whereas the integration properties of vestibular neurons differ significantly in vivo and in vitro [38]. Thus, further studies involving in vivo intracellular recordings from single primate central vestibular neurons are needed to uncover the mechanisms that mediate the observed nonlinearity.

### Consequences of Nonlinear Central Vestibular Processing for Higher Vestibular Pathways and Perception

During everyday activities, such as walking or running, the predominant frequencies of head rotation and translation are within 0.6–10 Hz in both humans [39]–[41] and monkeys [14],[42]. While significant harmonics up to 15–20 Hz can be present, their magnitude is generally <5% of the power found in the predominant frequency range. Taken together, these findings indicate that while active head movements cover a wide range of frequencies, most stimulation occurs at relatively low frequencies. This then leads to the question: What is the functional significance of nonlinear integration of afferent input by central vestibular neurons leading to attenuated responses to the low frequency components of self-motion?

One possibility is that the relative enhancement of high frequency power serves to effectively “whiten” (i.e., flatten) the output power spectrum of sensory neurons during everyday activities. For example, in vision, natural scenes are typically described by a spatial frequency amplitude spectrum that decreases as 1/frequency—or equivalently as a power spectrum that decreases as 1/frequency^2^ [43],[44]. A widespread view is that early visual neurons are tuned in such a way as to compensate for this decrease. Indeed, whitening would serve to equalize the neural responses across frequencies as originally proposed by Field [43]. Specifically, a neuron tuned to high frequencies would require an increased response gain to produce the same response as a neuron tuned to low frequencies (reviewed in [45],[46]). This mechanism bears a striking resemblance to preferential encoding of high frequency stimuli by central vestibular neurons demonstrated in the present study. Another possible mechanism that has been proposed to underlie whitening in the visual system is decorrelation [47], which includes neurons with bandpass tuning curves for which a portion of the curve rises with frequency. This latter model is not a likely candidate strategy for early vestibular processing since vestibular afferents and central neurons are characterized by high-pass rather than band-pass tuning.

Another possibility, which relates to the argument above, is that neuronal responses optimize our ability to reflexively respond to transient unexpected events. In particular, central vestibular neurons make descending projections to the spinal cord and mediate the vestibulo-spinal reflexes that ensure stable posture [9]. We note that, to date, the vestibular stimuli experienced during voluntary activities such as walking and running have primarily been quantified while subjects locomoted “in place” [39]. However, these studies might have underestimated the frequency content of natural vestibular stimuli. Indeed, higher frequency stimuli are experienced during natural locomotion since heel strikes can produce vibrations with frequencies as high as 75 Hz [48]. It is likely that these high frequency components are filtered out as the vibration passes up through the body. Thus, the enhanced neural responses to high frequency motion could be an effective coding strategy for countering the biomechanical filtering properties of the body segments during unexpected postural perturbations. Indeed, recent studies have demonstrated such frequency-specific filtering of vestibular-evoked postural responses in humans [49]. It is also noteworthy that central vestibular neurons are also much less responsive to active than passive motion [50],[51]. Accordingly, their response selectivity is likely to optimize our ability to reflexively respond to unexpected transient events. For example, if standing while riding the metro, or walking/running, one is likely to experience sudden stops or unexpected motion for which it is vital to generate compensatory postural reflexes.

Yet another possibility is that the nonlinear responses of central vestibular neurons constitute an adaptation mechanism that preserves the coding of both low and high frequency components of self-motion by preventing rectification (i.e., a complete cessation of firing). Specifically, such adaptation would serve to enhance the coding range by allowing responses to higher stimulus intensities through gain control. Gain control has been widely observed across systems and can be caused by multiple mechanisms [52]–[55]. Further studies that focus on how central vestibular neurons adapt to changes in natural self-motion stimuli are needed to investigate this possibility.

Finally, the central vestibular neurons that were the focus of the present study make contributions to higher-order vestibular processing including the computation of self-motion perception, spatial orientation (reviewed in [56]). However, to date, prior studies of self-motion perception [57] have focused on responses to motion containing frequencies <5 Hz and thus have only probed the lower portion of the physiologically relevant frequency range (i.e., 0–20 Hz) [14]. Accordingly, it is unlikely that the nonlinearities observed in the present study would have been significantly evoked in these studies. Interestingly, several studies have reported that perceptual responses to low frequency vestibular input are enhanced by a network property, termed velocity storage, which functions to lengthen the time constant of the vestibulo-ocular reflex [58]–[60]. This mechanism is mediated via reciprocal connections between the vestibular cerebellum and nuclei, and its dynamics are encoded in the responses of single central neurons. Our results predict that central neurons would exhibit dynamics consistent with velocity storage but that the amplitude of this effect should be reduced when low and high frequency stimuli are applied concurrently. Future experiments will be needed to investigate how the response selectivity of central vestibular neurons shapes postural responses as well as the perception of self-motion and spatial orientation.

### The Emergence of Feature Extraction: Function and General Principles Across Systems

As an alternative to the whitening hypothesis mentioned above, theoretical studies suggest that a common underlying principle of sensory processing is that the representation of information becomes more efficient in higher brain centers because neurons in these areas respond more selectively to specific features of natural sensory stimuli. This principle, commonly referred to as “sparse coding,” has been investigated in different sensory systems (see [4] for a review). Some of the most compelling evidence for a sparse code comes from experiments using stimuli resembling those which would be encountered during natural vision in primary visual cortex [61] and area V4 [62]. Parallel findings in the auditory [63], somatosensory [64], and olfactory [65] systems have provided further evidence that sensory processing is generally characterized by an increase in sparseness at higher levels. Here we focused on understanding the mechanisms underlying integration of afferent input by central vestibular neurons. While the linear filtering properties of central vestibular neurons and afferents were similar, confirming our previous results [10], we have shown here that a static nonlinearity causes a decreased response to low frequency stimuli in the presence of high frequency stimuli in central vestibular neurons but not afferents. We propose that this decreased response to the low frequency components of self-motion corresponds to feature detection in that it enables central vestibular neurons to respond selectively to the high frequency components. This is consistent with our previous results showing that individual central vestibular neurons transmit less information about the detailed time course of the stimulus than individual afferents [10]. We suggest that this enhanced feature selectivity displayed by central vestibular neurons could constitute a signature of sparse coding and that further sparsening occurs at subsequent levels of processing.

Our findings also suggest the intriguing possibility that central vestibular neurons implement gain control through divisive normalization, similar to that previously shown to occur in the visual [66], auditory [67], and olfactory [68] systems (see [69] for a review). In sensory systems for which neurons are tuned to different features of complex natural stimuli, divisive normalization provides an efficient nonlinear coding strategy that can reduce dependencies between stimulus features. Specifically, when multiple features are present in a given stimulus, the activity of a neuron tuned to a given feature is obtained by normalizing the response to that feature presented in isolation by the summed activity of neighbouring neurons tuned to the other features. As a result, an advantage is that divisive normalization effectively implements sensory gain control such that the neural response to a given feature is adaptively attenuated when other features are present. The attenuated response to low frequency head rotations that we observed in central vestibular neurons when these are presented concurrently with high frequency head rotations could be a signature of divisive normalization. Further studies are, however, needed to fully test this hypothesis and to understand the functional implications of the relatively negligible attenuation that was seen for high frequency stimulation.

Finally, our results provide evidence for a nonlinear mechanism that enables the preferential attenuation of the response to a given stimulus when multiple stimuli are presented at the same time. Such responses to stimuli consisting of sums of low and high frequency components are also seen in other systems and may thus be a general feature of sensory processing. For example, simultaneous masking presents some similarities with the effect described here as the presence of a high frequency sound can significantly degrade the perception of a low frequency sound [70]–[72]. Further, non-classical receptive field stimulation can strongly attenuate the responses to low but not high frequency input [61],[73]. We hypothesize that mechanisms similar to those described here might mediate these effects in other systems.

## Materials and Methods

Three macaque monkeys (two *Macaca mulatta* and one *Macaca fascicularis*) were prepared for chronic extracellular recording using aseptic surgical techniques [10],[74],[75]. All procedures were approved by the McGill University Animal Care Committee and were in compliance with the guidelines of the Canadian Council on Animal Care.

### Data Acquisition

The experimental setup and methods of data acquisition have been previously described for both vestibular afferents [18],[19],[76] and vestibular nuclei neurons [10],[51]. We used standard techniques to perform single unit recordings from 18 vestibular afferents [10],[76],[77] that innervate the horizontal semicircular canals and 21 vestibular-only (VO) neurons [10],[51],[74] in the medial vestibular nuclei that were sensitive to horizontal rotations. Resting discharge regularity in afferents was quantified by the normalized coefficient of variation (CV*) [10],[78]. Vestibular afferents with a CV*<0.15 were classified as regular, whereas those with a CV*≥0.15 were classified as irregular as done previously [18],[19],[79]. As such, five afferents were classified as regular and the remaining 13 were classified as irregular. VO neurons were classified as either type I or type II depending on whether they are excited or inhibited by rotations towards the ipsilateral side, respectively [80]. Nine VO neurons were type I and 12 were type II. Data from both groups were pooled as no notable difference was observed when quantifying their responses to the stimuli used here (unpublished data).

### Experimental Design

We used two classes of head velocity stimuli to characterize the responses of vestibular afferents and central neurons to horizontal rotations. The first class of stimuli consisted of noise stimuli characterized by a Gaussian distribution of angular velocities with zero mean and standard deviation (SD) of 20°/s each lasting 80 s. We used four different noise stimuli whose frequency content spanned the frequency range of natural vestibular stimuli (0–20 Hz) [14]: (1) low-pass filtered Gaussian white noise (8^th^ order Butterworth, 5 Hz cutoff frequency), henceforth referred to as the low frequency noise stimulus; (2) band-pass filtered Gaussian white noise (4^th^ order Butterworth, 15–20 Hz band), henceforth referred to as the high frequency noise stimulus; (3) the linear sum of the low and high frequency noise stimuli; and (4) low-pass filtered Gaussian white noise (8^th^ order Butterworth, 20 Hz cutoff frequency), henceforth referred to as the broadband noise stimulus. Our noise stimulation protocol consisted of the low frequency stimulus by itself, then the high frequency stimulus by itself, then the linear sum of the two, and finally the broadband noise stimulus.

The second class of stimuli consisted of single frequency sinusoidal rotations each lasting 80 s of amplitude 15°/s and frequencies 3 Hz and 17 Hz, henceforth referred to as the low and high frequency sinusoidal stimuli, respectively. These frequencies were chosen because they span the frequency range of natural vestibular stimuli (0–20 Hz) [14]. Our stimulation protocol consisted of delivering the low frequency sinusoidal stimulus, then the high frequency sinusoidal stimulus, and then the linear sum of the two.

### Traditional Linear System Analysis

For the analysis of responses to sinusoidal stimuli *s(t)*, the spike train from each neuron was converted into a binary sequence *r(t)* with a bin width of 1 ms. The value of any given bin was set to 1 if it contained an action potential and 0 otherwise, as done previously [18]. This binary sequence was then convolved with a Kaiser window with cutoff frequency 0.1 Hz above the stimulus frequency to obtain an estimate of the time dependent firing rate *f_measured_(t)* [81],[82]. The response gain was then computed by fitting a first order model f_fit_(t) = b+g * s(t−*t_d_)* to the data. Here *b* is the bias, *g* is the gain, and *t_d_* is the latency, respectively. We used a least squares regression to find the best fit parameter values that provide the maximum variance accounted for (VAF) given by *1*−*[var[f_fit_(t)*−*f_measured_(t)]/var(f_measured_(t))].* Here *var* is the variance and *f_measured_(t)* represents the actual firing rate [50],[74].

For noise stimuli, the stimulus waveform *s(t)* was also sampled with timesteps of 1 ms. The response sensitivity was computed from the gain *G(f) = |P_sr_(f)/P_ss_(f)|*, where *P_sr_(f)* is the cross-spectrum between the stimulus *s(t)* and binary sequence *r(t)*, and *P_ss_(f)* is the power spectrum of the stimulus *s(t).* All spectral quantities (i.e., power-spectra and cross-spectra) were estimated using multitaper techniques with 8 Slepian functions [83]. Estimates of gain for low (0–5 Hz) and high (15–20 Hz) frequencies were obtained by averaging the gain curves *G(f)* between 0 and 5 Hz and between 15 and 20 Hz, respectively.

### Coherence Measures

We also used the coherence function to measure the neural response to the noise stimuli used in this study. The coherence is defined by:
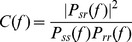
(1)Here *P_rr_(f)* is the power spectrum of the response *r(t)*. Based on the number of trials and tapers used in this study, the confidence limit for the magnitude of the coherence being significantly different from zero at the *p* = 0.05 level is 0.097 [83],[84] and all neurons in our dataset displayed maximum coherence values that were greater than 0.097 for at least one of the stimulation protocols.

It is important to note that, unlike the sensitivity *G(f)*, the coherence is based on the signal-to-noise ratio *SNR(f) = C(f)/[1*−*C(f)]* and thus takes neural variability into account [16]. As such, measuring the response using gain and coherence measures can sometimes give qualitatively different results [17],[18],[85]. The coherence is also related to a lower bound on the mutual information [86] that measures the amount of information that can be decoded linearly [87].

### Stationarity

We tested that the neural responses to both sinusoidal and noise stimuli were stationary in the following way. We divided each recorded neural response *r(t)* into 4 epochs of length 20 s and computed the mean firing rate, gain, and coherence in each epoch. We found that these did not differ significantly from one another for all neurons in our dataset and all stimuli (*p*>0.05, one-way ANOVAs).

### Normalization

All gain and coherence measures were normalized in the following way. The curves in response to the high frequency stimuli (noise or sinusoidal) were normalized by their values at 17 Hz. The curves in response to low frequency stimuli were also normalized by these values. The curves obtained in response to the sum of the low and high frequency stimuli were normalized by their values at 17 Hz.

### Attenuation

We quantified response gain attenuation by:
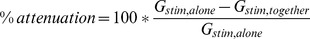
(2)where G_stim,alone_ is the gain in response to stimulus “stim” when it is presented by itself and G_stim,together_ is the gain in response to stimulus “stim” when it is presented concurrently with another stimulus. We also quantified coherence response attenuation by:
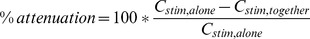
(3)where C_stim,alone_ is the coherence in response to stimulus “stim” averaged over the stimulus's frequency range when it is presented by itself and C_stim,together_ is the coherence in response to stimulus “stim” averaged over the stimulus's frequency range when it is presented concurrently with another stimulus.

### Input-Output Relationships

We quantified the output as the time varying firing rate, which was obtained by filtering the response r(t) using a Kaiser filter with cutoff frequency 5 Hz above the highest frequency contained in the stimulus input [81]. We then computed the cross-correlation function between the filtered response and the horizontal head velocity stimulus s(t) and noted the lag at which it was maximal. This lag was then used to align the response r(t) with the stimulus s(t). We then plotted r(t) as a function of s(t) and took the average of values in bins of 1 deg/s. To quantify whether these curves were well-fit by a straight line, we performed a linear least-squares fit over the range 10 to 20 deg/s and computed *R* ^2^ over the range −30 to −20 deg/s.

### Rescaled Input-Output Relationships

We rescaled input-output relationships in order to plot the output firing rate of VO neurons as a function of the input afferent firing rate. Because central vestibular neurons receive input from a heterogeneous population of afferents, we estimate the afferent input firing rate in the following manner. First, we took the average gain curves of regular and irregular afferents as a function of frequency obtained by Sadeghi et al. [19] since this corresponds, to the best of our knowledge, to the largest dataset on primate vestibular afferents. We then fit these curves using the following expression [20],[88],[89]:
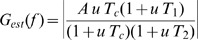
(4)where u = 2 π i f. Here T_c_ and T_2_ are the long and short time constants of the torsion–pendulum model of canal biomechanics and T_1_ is proportional to the ratio of acceleration to velocity sensitivity of the afferent response. Similar models have more recently been shown to provide an accurate description of canal afferent responses in monkeys [79],[90] up to 20 Hz [91], in chinchillas [88],[92] and mice [93]. We used A = 0.428 (spk/s)/(deg/s), T_1_ = 0.015 s, T_2_ = 0.003 s, and T_c_ = 5.7 s to fit the average gain curve for regular vestibular afferents [20]. A was adjusted to match the data of Sadeghi et al. [19] under control conditions. To fit the average gain curve of irregular afferents, we used A = 0.765 (spk/s)/(deg/s), T_1_ = 0.0085 s, T_2_ = 0.003 s, and T_c_ = 5.7 s. A and T_1_ were adjusted to match the average gain curve for C and D-irregulars from Sadeghi et al.'s [19] data under control conditions since C and D-irregulars were encountered with roughly equal probability [19].

The input afferent firing rate is then given by:
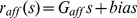
(5)

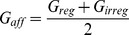
(6)where G_reg_ and G_irreg_ are the gains of regular and irregular afferents averaged over the stimulus's frequency content, respectively, and *G_aff_* is the average between the two values. We took the average since about 50% of afferents encountered were regular and the other 50% were irregular in Sadeghi et al.'s [19] dataset. We used a *bias* of 104.30 spk/s, which corresponds to the average baseline firing rate of the afferent population observed experimentally [19].

### Model

Our model assumes that VO neurons display a static input-output relationship with respect to their afferent input. We estimated this relationship by fitting a 6^th^ order polynomial to the input-output relationship obtained experimentally with the high frequency noise stimulus. As a result, the output firing rate of the VO neuron is given by:
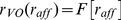
(7)where *r_VO_* is the VO neuron's firing rate, *r_aff_* is the afferent firing rate, and *F* is the estimate of the static input-output relationship.

We now consider the input *s* to consist of two stimuli. We will refer to one stimulus as the “signal” and to the other as the “masker.” Note that, while the terms “signal” versus “masker” are arbitrary, this division allows us to focus on the coding of one input (i.e., the input designated as the signal).

The VO neuron's response to the signal and masker stimuli is then given by:
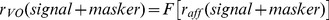
(8)

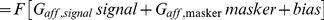
(9)where *G_aff,signal_* and *G_aff,masker_* are the afferent gains to the signal and masker, respectively. These are obtained by averaging the afferent gains over the signal and masker's frequency contents, respectively. In order to obtain the VO neuron's firing rate as a function of the signal alone, it is necessary to average over the distribution of values that can be taken by the masker. As signal and masker are not correlated, this distribution is equal to the probability distribution of the masker, which is taken to be normal with mean 0 and standard deviation *σ_masker_*, thus:
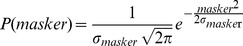
(10)The VO neuron's firing rate is then given by:
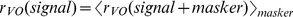
(11)

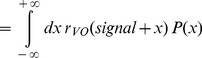
(12)where *x* is the masker. The integral was evaluated numerically using a Riemann sum approximation with binwidth 1 deg/s. This model can then be used to predict the VO neuron's input-output relationship when arbitrary signal and masker stimuli are used. In order to get some intuition, we expanded F into a Taylor series in equation (12) to obtain:
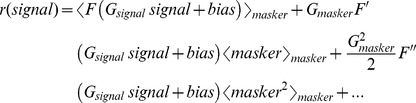
(13)where F′ and F″ are the first and second derivatives of F, respectively. The first term simply corresponds to the firing rate when no masker is present (i.e., 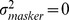) and the term 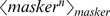
is equal to the n^th^ order moment of the Gaussian distribution *P(masker)*. In particular, all moments for n odd are equal to zero (this comes from the fact that the distribution is symmetric with respect to its mean) while the second moment is simply equal to the variance 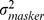. Neglecting all higher order moments gives:
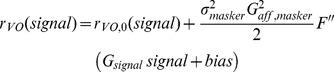
(14)where *r* *_VO_*(*signal*) is the VO neuron's firing rate for a given value of the signal in the presence of the masker and *r* *_VO,_*_0_(*signal*) is the firing rate for the same value of the signal when the masker is absent (i.e., 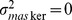). Inspection of equation (14) shows that the masker has no effect on the output firing rate *r* *_VO_*(*signal*) if *F* is a linear function, as we then have *F*″(x) = 0 for any x. Further, the sign of the correction depends solely on the sign of the second derivative since all other terms are positive. As such, the masker will increase the average firing rate in response to the signal in regions where *F* is convex and decrease it in regions where *F* is concave. The amount by which the firing rate increases/decreases grows in magnitude with the masker variance 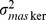 but also depends on the gain of the afferents to the masker *G_aff, masker_*. Since the afferents display gains that increase as a function of frequency, maskers with higher frequency content will lead to a greater correction in firing rate than maskers with lower frequency content for a given variance 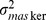. Equation (14) then allows us to evaluate the percentage attenuation in gain by taking its derivative and evaluating it at signal = 0 and substituting the result into equation (2):
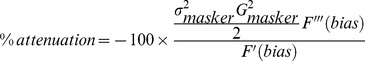
(15)where *F‴* is the third derivative of *F*.

### Linear Nonlinear Cascade Model

We used a linear-nonlinear (LN) cascade model [22] to characterize the response properties of both afferents and VO neurons to noise stimuli. This model predicts that a neuron's firing rate *r_predicted_* at any instant is a function f of the linear firing rate r_linear_ plus the baseline firing rate r_bias_. The linear firing rate is obtained by convolving the stimulus with the optimal linear filter *H(t)*. Thus, we have:
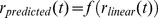
(16)

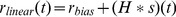
(17)where “*” denotes the convolution operation and H(t) is the inverse Fourier transform of the transfer function 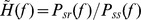. We estimated f by plotting the actual firing rate *r(t)*, which was computed as described above, as a function of the linear prediction *r_linear_* [22]. To quantify whether these curves were well-fit by a straight line, we performed a linear least-squares fit over the ranges 80–120 and 100–140 spk/s for central VO neurons and afferents, respectively. We then computed the *R* ^2^ over the ranges −17–120 and 20–140 spk/s for central VO neurons and afferents, respectively. In practice, *H(t)*, *r_bias_*, and f were all computed using the first half of the recorded activity for a given neuron. We then compared the predicted firing rate *r_predicted_(t)* computed using equation (16) against the actual firing rate *r(t)* for the second half of the recorded activity and quantified the goodness-of-fit of the LN model by computing *R* ^2^.

### Statistics

Values are reported as mean ± STD in the text. Error bars or gray bands represent 1 SEM. Throughout, “**” and “*” indicate statistical significance using a paired *t* test at the *p* = 0.01 and *p* = 0.05 levels, respectively. “NS” indicates that the *p* value was above 0.05.

## Supporting Information

Figure S1 Central VO neurons but not afferents respond nonlinearly to sums of low and high frequency noise stimuli as quantified by coherence measures. (A, B) Coherence curves as a function of frequency for an example VO neuron (A) and averaged over the population (B). (C) Population-averaged average normalized coherence values for central VO neurons. (D, E) Coherence curves as a function of frequency for an example regular afferent (D) and averaged over the population (E). (F) Population-averaged average normalized coherence values for regular afferents. (G, H) Coherence curves as a function of frequency for an example irregular afferent (G) and averaged over the population (H). (I) Population-averaged average normalized coherence values for irregular afferents.
(TIF)

Figure S2 Central vestibular neurons respond nonlinearly to sums of sinusoidal stimuli. (A–C) Example central vestibular neuron responses to 3 Hz (A), 17 Hz (B), and 3+17 Hz (C) sinusoidal rotations. The insets show the power spectra of the input stimuli (black) and output firing rate (red and blue). (D, E) Comparison between the actual response and that predicted from a linear system for the same example neuron for the 3 Hz (D) and 17 Hz (E) components of 3+17 Hz stimulation. (F) Population-averaged normalized gains for central VO neurons. Note the gain for 3 Hz is strongly attenuated in the presence of 17 Hz (*p*<10^−3^, paired *t* test, *n* = 11). In contrast, the gain at 17 Hz was not significantly altered by simultaneously presenting the 3 Hz stimulus (*p* = 0.97, paired *t* test, *n* = 8). (G) Population-averaged percentage attenuation at low (3 Hz) and high (17 Hz) for central neurons. The firing rate estimates were obtained by convolving the spike trains with a Kaiser filter (see Materials and Methods).
(TIF)

Figure S3 Analysis of unfiltered spike trains confirms that central vestibular neurons respond nonlinearly to sums of sinusoidal stimuli. (A–C) Spike train power spectra for the same example central VO neuron shown in Figure S2 to 3 Hz (A), 17 Hz (B), and 3+17 Hz (C) sinusoidal rotations. Note that the power at 3 Hz was lower for 3+17 Hz than for 3 Hz stimulation and that the power at 17 Hz for 17 Hz stimulation was similar to that for 3+17 Hz stimulation.
(TIF)

Figure S4 Central VO neurons as well as afferents do not show rectification and/or saturation when stimulated by the low and high frequency head rotations used in this study. (A–C) Phase histograms for an example VO neuron (A), regular afferent (B), and irregular afferent (C). The solid curves show the best sinusoidal fits. The dashed lines indicate the mean firing rates. Note that in no case do the histograms display either saturation or rectification. The population-averaged percentage of bins in the phase histograms corresponding to values less than 5% of the mean was 0 in more than 98% of cases, indicating no significant rectification. This was also true for 3 Hz and 3+17 Hz sinusoidal rotation (unpublished data) and for all neurons in the population. The population-averaged Variance-Accounted-For (VAF) of the sinusoidal fit for all three types of neurons was not significantly different between the different sinusoidal stimuli (*p*>0.15, *t* tests). This was also true for the noise stimuli (unpublished data).
(TIF)

Figure S5 Afferents display a linear relationship between output firing rate and input head velocity. (A) Population-averaged firing rate as a function of head velocity for regular afferents when the low frequency (0–5 Hz) noise stimulus was applied in isolation (blue) and concurrently with the high frequency (15–20 Hz) noise stimulus (black). Inset: firing rate as a function of head velocity for an example regular afferent. (B) Population-averaged firing rate as a function of head velocity for regular afferents when the high-frequency (15–20 Hz) noise stimulus was applied in isolation (red) and concurrently with the low frequency (0–5 Hz) noise stimulus (dashed black). Inset: firing rate as a function of head velocity for the same regular afferent. (C) Population-averaged firing rate as a function of head velocity for irregular afferents when the low frequency (0–5 Hz) noise stimulus was applied in isolation (blue) and concurrently with the high frequency (15–20 Hz) noise stimulus (black). Inset: firing rate as a function of head velocity for an example irregular afferent. (C) Population-averaged firing rate as a function of head velocity for irregular afferents when the high-frequency (15–20 Hz) noise stimulus was applied in isolation (red) and concurrently with the low frequency (0–5 Hz) noise stimulus (dashed black). Inset: firing rate response as a function of head velocity for the same irregular afferent.
(TIF)

Figure S6 Individual central neurons display nonlinear responses. (A) Firing rate as a function of head velocity for an example central VO neuron when the low frequency (0–5 Hz) noise stimulus was applied in isolation (blue) and concurrently with the high frequency (15–20 Hz) noise stimulus (black). Both curves were well fit by straight lines (dashed lines). (B) Firing rate as a function of head velocity for the same example central VO neuron when the high frequency (15–20 Hz) noise stimulus was applied in isolation (red) and concurrently with the low frequency (0–5 Hz) noise stimulus (long dashed black). Note that both curves were not well fit by straight lines (short dashed lines).
(TIF)

Figure S7 Characterization of central VO neurons and afferents by LN cascade models. (A) Actual firing rate as a function of the linear prediction for an example regular afferent. Inset: the filter *H(t)* for this same afferent. (B) Population-averaged actual firing rate as a function of the linear prediction for regular afferents. Inset: population-averaged filter *H(t)* for regular afferents. (C) Actual firing rate as a function of the linear prediction for an example irregular afferent. Inset: the filter *H(t)* for this same afferent. (D) Population-averaged actual firing rate as a function of the linear prediction for irregular afferents. Inset: population-averaged filter *H(t)* for irregular afferents. (E) Actual firing rate as a function of the linear prediction for an example central VO neuron. Inset: the filter *H(t)* for this same VO neuron. (F) Population-averaged actual firing rate as a function of the linear prediction for central VO neurons. Inset: population-averaged filter *H(t)* for central VO neurons. Throughout, the identity line is shown in green.
(TIF)

Figure S8 LN analysis reveals that central vestibular neurons but not afferents display a static nonlinearity in response to different self-motion stimuli. (A) Population-averaged actual firing rate as a function of the linear prediction for afferents in response to 0–20 Hz noise (green), 0–5 Hz noise (blue), 15–20 Hz noise (red), and 0–5 Hz+15–20 Hz noise (black). Note that all the curves are linear and overlap but that the blue curve extends over a narrower range than all the others. All the curves were further well fit by straight lines (*R* ^2^ = 0.99 in all cases). (B) Population-averaged actual firing rate as a function of the linear prediction for central VO neurons in response to 0–20 Hz noise (green), 0–5 Hz noise (blue), 15–20 Hz noise (red), and 0–5 Hz+15–20 Hz noise (black). Note that all the curves overlap but that the blue curve extends over a narrower range than all the others. As such, the blue curve is relatively better fit by a straight line (0–5 Hz: *R* ^2^ = 0.91; 15–20 Hz: *R* ^2^ = 0.58; 0–5 Hz+15–20 Hz: *R* ^2^ = 0.37; 0–20 Hz: *R* ^2^ = 0.62).
(TIF)

## Abbreviations

CV: coefficient of variation
LN: linear-nonlinear
STD: standard deviation
SEM: standard error of the mean
VO: vestibular-only, VN, vestibular nuclei

## References

[1] Van Essen D. C, Anderson C. H, Felleman D. J (1992). Information processing in the primate visual system: an integrated systems perspective.. Science.

[2] Barlow H. B (1972). Single units and sensation: a neuron doctrine for perceptual psychology.. Perception.

[3] Rolls E. T, Tovee M. J (1995). Sparseness of the neuronal representation of stimuli in the primate temporal visual cortex.. J Neurophysiol.

[4] Olshausen B. A, Field D. J (2004). Sparse coding of sensory inputs.. Curr Opinion in Neurobiol.

[5] Hromadka T, Deweese M. R, Zador A. M (2008). Sparse representation of sounds in the unanesthetized auditory cortex.. PLoS Biol.

[6] Attwell D, Laughlin S. B (2001). An energy budget for signaling in the grey matter of the brain.. J Cereb Blood Flow Metab.

[7] Földiák P, Young M. P, Arbib M. A (1995). Sparse coding in the primate cortex.. The handbook of brain theory and neural networks.

[8] Goldberg J. M (2000). Afferent diversity and the organisation of central vestibular pathways.. Exp Brain Res.

[9] Cullen K. E, Roy J. E (2004). Signal processing in the vestibular system during active versus passive head movements.. J Neurophysiol.

[10] Massot C, Chacron M. J, Cullen K. E (2011). Information transmission and detection thresholds in the vestibular nuclei: single neurons versus population encoding.. J Neurophysiol.

[11] Bagnall M. W, McElvain L. E, Faulstich M, du Lac S (2008). Frequency-independent synaptic transmission supports a linear vestibular behavior.. Neuron.

[12] Robinson D. A (1976). Adaptive gain control of vestibuloocular reflex by the cerebellum.. J Neurophysiol.

[13] Pulaski P. D, Zee D. S, Robinson D. A (1981). The behavior of the vestibulo-ocular reflex at high velocities of head rotation.. Brain Res.

[14] Huterer M, Cullen K. E (2002). Vestibuloocular reflex dynamics during high-frequency and high-acceleration rotations of the head on body in rhesus monkey.. J Neurophysiol.

[15] Minor L. B, Lasker D. M, Backous D. D, Hullar T. E (1999). Horizontal vestibuloocular reflex evoked by high-acceleration rotations in the squirrel monkey. I. Normal responses.. J Neurophysiol.

[16] Stein R. B, Gossen E. R, Jones K. E (2005). Neuronal variability: noise or part of the signal?. Nat Rev Neurosci.

[17] Chacron M. J, Maler L, Bastian J (2005). Electroreceptor neuron dynamics shape information transmission.. Nat Neurosci.

[18] Sadeghi S. G, Chacron M. J, Taylor M. C, Cullen K. E (2007). Neural variability, detection thresholds, and information transmission in the vestibular system.. J Neurosci.

[19] Sadeghi S. G, Minor L. B, Cullen K. E (2007). Response of vestibular-nerve afferents to active and passive rotations under normal conditions and after unilateral labyrinthectomy.. J Neurophysiol.

[20] Fernandez C, Goldberg J. M (1971). Physiology of peripheral neurons innervating semicircular canals of the squirrel monkey. II. Response to sinusoidal stimulation and dynamics of peripheral vestibular system.. J Neurophysiol.

[21] de la Rocha J, Doiron B, Shea-Brown E, Josic K, Reyes A (2007). Correlation between neural spike trains increases with firing rate.. Nature.

[22] Chichilnisky E. J (2001). A simple white noise analysis of neuronal light responses.. Network.

[23] Ermentrout B (1998). Linearization of F-I curves by adaptation.. Neural Comput.

[24] Doiron B, Longtin A, Berman N, Maler L (2001). Subtractive and divisive inhibition: effect of voltage-dependent inhibitory conductances and noise.. Neural Computation.

[25] Stemmler M (1996). A single spike suffices: the simplest form of stochastic resonance in model neurons.. Network.

[26] Schneider A. D, Cullen K. E, Chacron M. J (2011). In vivo conditions induce faithful encoding of stimuli by reducing nonlinear synchronization in vestibular sensory neurons.. PLoS Comput Biol.

[27] Wark B, Lundstrom B. N, Fairhall A (2007). Sensory adaptation.. Curr Opin Neurobiol.

[28] Musallam S, Tomlinson R. D (2002). Asymmetric integration recorded from vestibular-only cells in response to position transients.. J Neurophysiol.

[29] Newlands S. D, Lin N, Wei M (2009). Response linearity of alert monkey non-eye movement vestibular nucleus neurons during sinusoidal yaw rotation.. J Neurophysiol.

[30] Sadeghi S. G, Goldberg J. M, Minor L. B, Cullen K. E (2009). Effects of canal plugging on the vestibuloocular reflex and vestibular nerve discharge during passive and active head rotations.. J Neurophysiol.

[31] Broussard D. M (2009). Dynamics of glutamatergic synapses in the medial vestibular nucleus of the mouse.. Eur J Neurosci.

[32] Ris L, Hachemaoui M, Vibert N, Godaux E, Vidal P. P (2001). Resonance of spike discharge modulation in neurons of the guinea pig medial vestibular nucleus.. J Neurophysiol.

[33] Popratiloff A, Peusner K. D (2007). Otolith fibers and terminals in chick vestibular nuclei.. J Comp Neurol.

[34] Malinvaud D, Vassias I, Reichenberger I, Rossert C, Straka H (2010). Functional organization of vestibular commissural connections in frog.. J Neurosci.

[35] Wulff P, Schonewille M, Renzi M, Viltono L, Sassoe-Pognetto M (2009). Synaptic inhibition of Purkinje cells mediates consolidation of vestibulo-cerebellar motor learning.. Nat Neurosci.

[36] Straka H, Vibert N, Vidal P. P, Moore L. E, Dutia M. B (2005). Intrinsic membrane properties of vertebrate vestibular neurons: function, development and plasticity.. Prog Neurobiol.

[37] Beraneck M, Pfanzelt S, Vassias I, Rohregger M, Vibert N (2007). Differential intrinsic response dynamics determine synaptic signal processing in frog vestibular neurons.. J Neurosci.

[38] Schneider A. D, Cullen K. E, Chacron M. J (2011). In vivo conditions induce faithful encoding of stimuli by reducing nonlinear synchronization in vestibular sensory neurons.. PLoS Comp Biol.

[39] Grossman G. E, Leigh R. J, Abel L. A, Lanska D. J, Thurston S. E (1988). Frequency and velocity of rotational head perturbations during locomotion.. Exp Brain Res.

[40] Pozzo T, Berthoz A, Lefort L (1990). Head stabilization during various locomotor tasks in humans. I. Normal subjects.. Exp Brain Res.

[41] Pozzo T, Berthoz A, Lefort L, Vitte E (1991). Head stabilization during various locomotor tasks in humans. II. Patients with bilateral peripheral vestibular deficits.. Exp Brain Res.

[42] Armand M, Minor L. B (2001). Relationship between time- and frequency-domain analyses of angular head movements in the squirrel monkey.. J Comput Neurosci.

[43] Field D. J (1987). Relations between the statistics of natural images and the response properties of cortical cells.. J Opt Soc Am A.

[44] Burton G. J, Moorhead I. R (1987). Color and spatial structure in natural scenes.. Appl Opt.

[45] Field D. J, Brady N (1997). Visual sensitivity, blur and the sources of variability in the amplitude spectra of natural scenes.. Vision Res.

[46] Graham D. J, Chandler D. M, Field D. J (2006). Can the theory of “whitening” explain the center-surround properties of retinal ganglion cell receptive fields?. Vision Res.

[47] Atick J. J, Redlich A (1992). What does the retina know about natural scenes.. Neural Comput.

[48] Simon S. R, Paul I. L, Mansour J, Munro M, Abernethy P. J (1981). Peak dynamic force in human gait.. J Biomech.

[49] Dakin C. J, Luu B. L, van den Doel K, Inglis J. T, Blouin J. S (2010). Frequency-specific modulation of vestibular-evoked sway responses in humans.. J Neurophysiol.

[50] Roy J. E, Cullen K. E (2001). Selective processing of vestibular reafference during self-generated head motion.. J Neurosci.

[51] Roy J. E, Cullen K. E (2004). Dissociating self-generated from passively applied head motion: neural mechanisms in the vestibular nuclei.. J Neurosci.

[52] Chance F. S, Abbott L. F, Reyes A. D (2002). Gain modulation from background synaptic input.. Neuron.

[53] Wark B, Lundstrom B. N, Fairhall A (2007). Sensory adaptation.. Curr Opin Neurobiol.

[54] Fairhall A. L, Lewen G. D, Bialek W, de Ruyter van Steveninck R. R (2001). Efficiency and ambiguity in an adaptive neural code.. Nature.

[55] Rothman J. S, Cathala L, Steuber V, Silver R. A (2009). Synaptic depression enables neuronal gain control.. Nature.

[56] Angelaki D. E, Cullen K. E (2008). Vestibular system: the many facets of a multimodal sense.. Annu Rev Neurosci.

[57] Grabherr L, Nicoucar K, Mast F. W, Merfeld D. M (2008). Vestibular thresholds for yaw rotation about an earth-vertical axis as a function of frequency.. Exp Brain Res.

[58] Okada T, Grunfeld E, Shallo-Hoffmann J, Bronstein A. M (1999). Vestibular perception of angular velocity in normal subjects and in patients with congenital nystagmus.. Brain.

[59] Bronstein A. M, Grunfeld E. A, Faldon M, Okada T (2008). Reduced self-motion perception in patients with midline cerebellar lesions.. Neuroreport.

[60] Bertolini G, Ramat S, Laurens J, Bockisch C. J, Marti S (2011). Velocity storage contribution to vestibular self-motion perception in healthy human subjects.. J Neurophysiol.

[61] Vinje W. E, Gallant J. L (2000). Sparse coding and decorrelation in primary visual cortex during natural vision.. Science.

[62] Carlson E. T, Rasquinha R. J, Zhang K, Connor C. E (2010). A sparse object coding scheme in area V4.. Curr Biol.

[63] DeWeese M. R, Wehr M, Zador A. M (2003). Binary spiking in auditory cortex.. J Neurosci.

[64] Brecht M, Sakmann B (2002). Dynamic representation of whisker deflection by synaptic potentials in spiny stellate and pyramidal cells in the barrels and septa of layer 4 rat somatosensory cortex.. J Physiol.

[65] Laurent G (2002). Olfactory network dynamics and the coding of multidimensional signals.. Nate Rev Neurosci.

[66] Heeger D. J (1992). Normalization of cell responses in cat striate cortex.. Vis Neurosci.

[67] Schwartz O, Simoncelli E. P (2001). Natural signal statistics and sensory gain control.. Nat Neurosci.

[68] Olsen S. R, Bhandawat V, Wilson R. I (2010). Divisive normalization in olfactory population codes.. Neuron.

[69] Carandini M, Heeger D. J (2012). Normalization as a canonical neural computation.. Nat Rev Neurosci.

[70] Gelfand S (2004). Hearing: an introduction to psychological and physiological acoustics.

[71] Gutschalk A, Micheyl C, Oxenham A. J (2008). Neural correlates of auditory perceptual awareness under informational masking.. PLoS Biol.

[72] Makous J. C, Friedman R. M, Vierck C. J (1995). A critical band filter in touch.. J Neurosci.

[73] Chacron M. J, Doiron B, Maler L, Longtin A, Bastian J (2003). Non-classical receptive field mediates switch in a sensory neuron's frequency tuning.. Nature.

[74] Sylvestre P. A, Cullen K. E (1999). Quantitative analysis of abducens neuron discharge dynamics during saccadic and slow eye movements.. J Neurophysiol.

[75] Sadeghi S. G, Minor L. B, Cullen K. E (2006). Dynamics of the horizontal vestibuloocular reflex after unilateral labyrinthectomy: response to high frequency, high acceleration, and high velocity rotations.. Exp Brain Res.

[76] Cullen K. E, Minor L. B (2002). Semicircular canal afferents similarly encode active and passive head-on-body rotations: implications for the role of vestibular efference.. J Neurosci.

[77] Lisberger S. G, Pavelko T. A (1986). Vestibular signals carried by pathways subserving plasticity of the vestibulo-ocular reflex in monkeys.. J Neurosci.

[78] Goldberg J. M, Smith C. E, Fernandez C (1984). Relation between discharge regularity and responses to externally applied galvanic currents in vestibular nerve afferents of the squirrel monkey.. J Neurophysiol.

[79] Haque A, Angelaki D. E, Dickman J. D (2004). Spatial tuning and dynamics of vestibular semicircular canal afferents in rhesus monkeys.. Exp Brain Res.

[80] Duensing F, Schaefer K. P (1958). [The activity of single neurons in the region of the vestibular nuclei in horizontal acceleration, with special reference to vestibular nystagmus].. Arch Psychiatr Nervenkr Z Gesamte Neurol Psychiatr.

[81] Cherif S, Cullen K. E, Galiana H. L (2008). An improved method for the estimation of firing rate dynamics using an optimal digital filter.. J Neurosci Methods.

[82] Oppenheim A. V, Schafer R. W (1989). Discrete-time signal processing.

[83] Jarvis M. R, Mitra P. P (2001). Sampling properties of the spectrum and coherency of sequences of action potentials.. Neural Comput.

[84] Berg R. W, Whitmer D, Kleinfeld D (2006). Exploratory whisking by rat is not phase locked to the hippocampal theta rhythm.. J Neurosci.

[85] Lindner B, Gangloff D, Longtin A, Lewis J. E (2009). Broadband coding with dynamic synapses.. J Neurosci.

[86] Borst A, Theunissen F (1999). Information theory and neural coding.. Nat Neurosci.

[87] Roddey J. C, Girish B, Miller J. P (2000). Assessing the performance of neural encoding models in the presence of noise.. J Comput Neurosci.

[88] Hullar T. E, Della Santina C. C, Hirvonen T, Lasker D. M, Carey J. P (2005). Responses of irregularly discharging chinchilla semicircular canal vestibular-nerve afferents during high-frequency head rotations.. J Neurophysiol.

[89] Sadeghi S. G, Minor L. B, Cullen K. E (2010). Multimodal integration after unilateral labyrinthine lesion: single vestibular nuclei neuron responses and implications for postural compensation.. J Neurophysiol.

[90] Minor L. B, Goldberg J. M (1991). Vestibular-nerve inputs to the vestibulo-ocular reflex: a functional-ablation study in the squirrel monkey.. J Neurosci.

[91] Ramachandran R, Lisberger S. G (2006). Transformation of vestibular signals into motor commands in the vestibuloocular reflex pathways of monkeys.. J Neurophysiol.

[92] Hullar T. E, Minor L. B (1999). High-frequency dynamics of regularly discharging canal afferents provide a linear signal for angular vestibuloocular reflexes.. J Neurophysiol.

[93] Lasker D. M, Han G. C, Park H. J, Minor L. B (2008). Rotational responses of vestibular-nerve afferents innervating the semicircular canals in the C57BL/6 mouse.. J Assoc Res Otolaryngol.

