# Supplementary figures and images for: The Vestibular System Implements a Linear–Nonlinear Transformation In Order to Encode Self-Motion
Source: PLoS Biol. 2012 Jul 24;10(7):e1001365. doi: 10.1371/journal.pbio.1001365 (PMC3404115; doi:10.1371/journal.pbio.1001365)

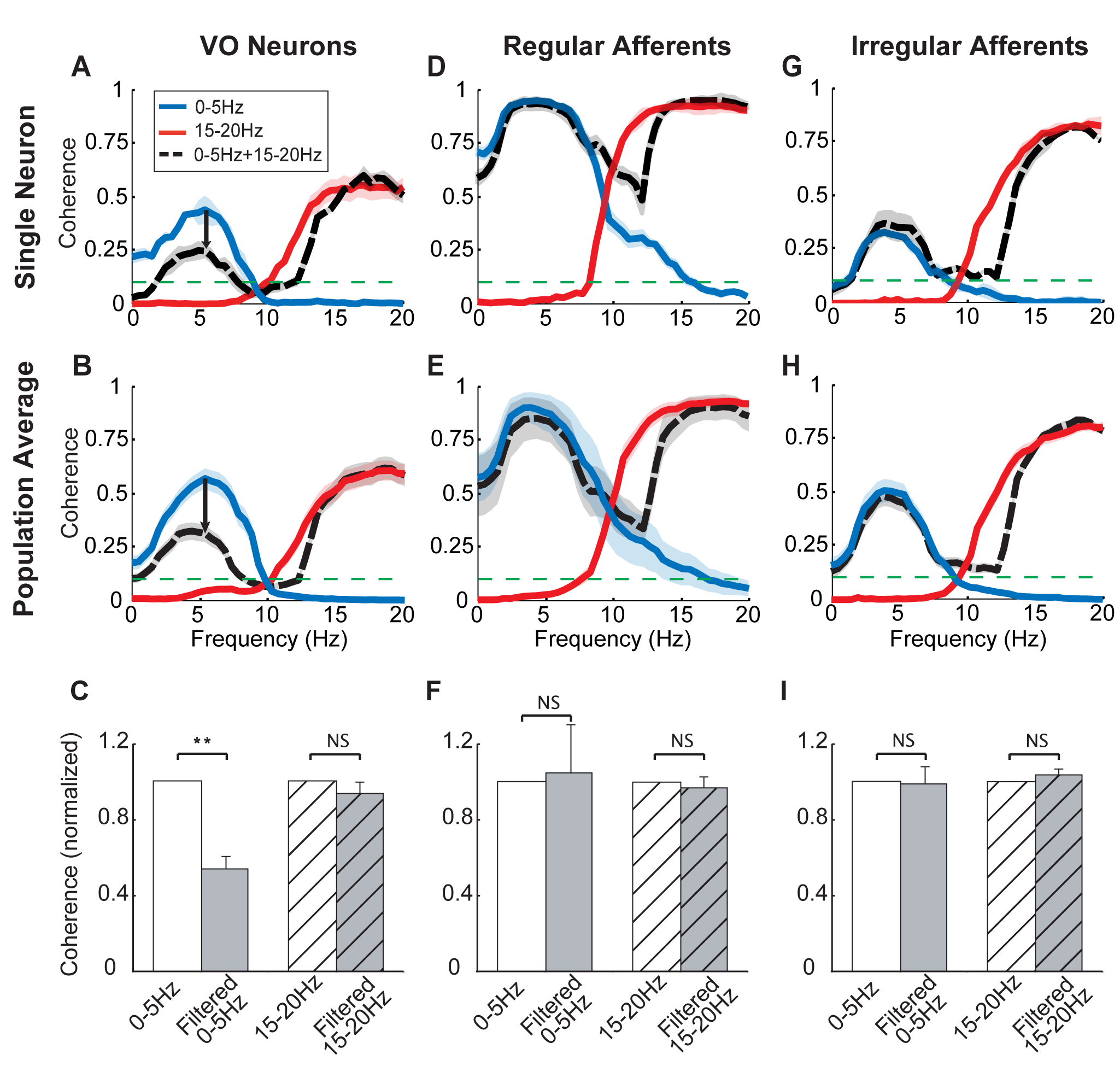

Supplement: Figure S1 — Central VO neurons but not afferents respond nonlinearly to sums of low and high frequency noise stimuli as quantified by coherence measures. (A, B) Coherence curves as a function of frequency for an example VO neuron (A) and averaged over the population (B). (C) Population-averaged average normalized coherence values for central VO neurons. (D, E) Coherence curves as a function of frequency for an example regular afferent (D) and averaged over the population (E). (F) Population-averaged average normalized coherence values for regular afferents. (G, H) Coherence curves as a function of frequency for an example irregular afferent (G) and averaged over the population (H). (I) Population-averaged average normalized coherence values for irregular afferents. (TIF) [file pbio.1001365.s001.tif]

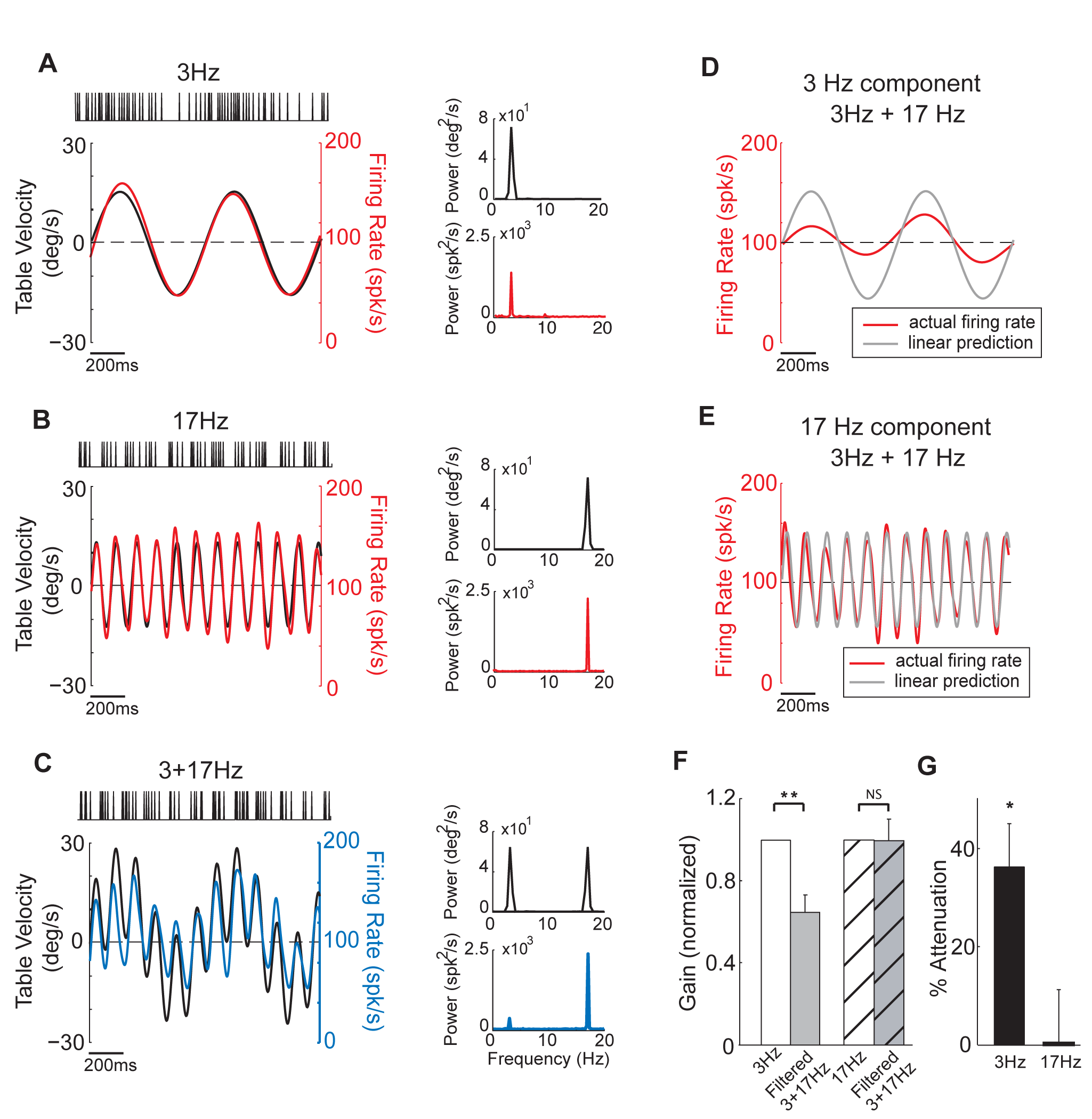

Supplement: Figure S2 — Central vestibular neurons respond nonlinearly to sums of sinusoidal stimuli. (A–C) Example central vestibular neuron responses to 3 Hz (A), 17 Hz (B), and 3+17 Hz (C) sinusoidal rotations. The insets show the power spectra of the input stimuli (black) and output firing rate (red and blue). (D, E) Comparison between the actual response and that predicted from a linear system for the same example neuron for the 3 Hz (D) and 17 Hz (E) components of 3+17 Hz stimulation. (F) Population-averaged normalized gains for central VO neurons. Note the gain for 3 Hz is strongly attenuated in the presence of 17 Hz (p<10−3, paired t test, n = 11). In contrast, the gain at 17 Hz was not significantly altered by simultaneously presenting the 3 Hz stimulus (p = 0.97, paired t test, n = 8). (G) Population-averaged percentage attenuation at low (3 Hz) and high (17 Hz) for central neurons. The firing rate estimates were obtained by convolving the spike trains with a Kaiser filter (see Materials and Methods). (TIF) [file pbio.1001365.s002.tif]

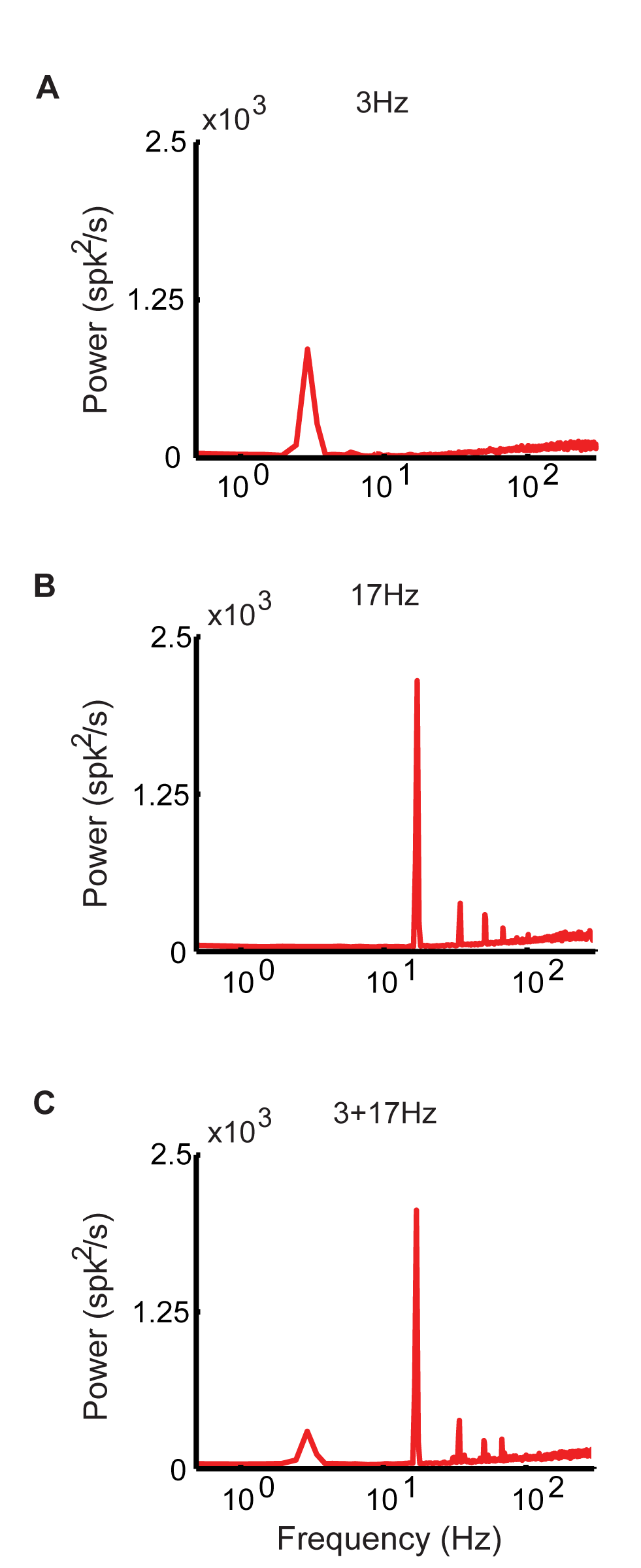

Supplement: Figure S3 — Analysis of unfiltered spike trains confirms that central vestibular neurons respond nonlinearly to sums of sinusoidal stimuli. (A–C) Spike train power spectra for the same example central VO neuron shown in Figure S2 to 3 Hz (A), 17 Hz (B), and 3+17 Hz (C) sinusoidal rotations. Note that the power at 3 Hz was lower for 3+17 Hz than for 3 Hz stimulation and that the power at 17 Hz for 17 Hz stimulation was similar to that for 3+17 Hz stimulation. (TIF) [file pbio.1001365.s003.tif]

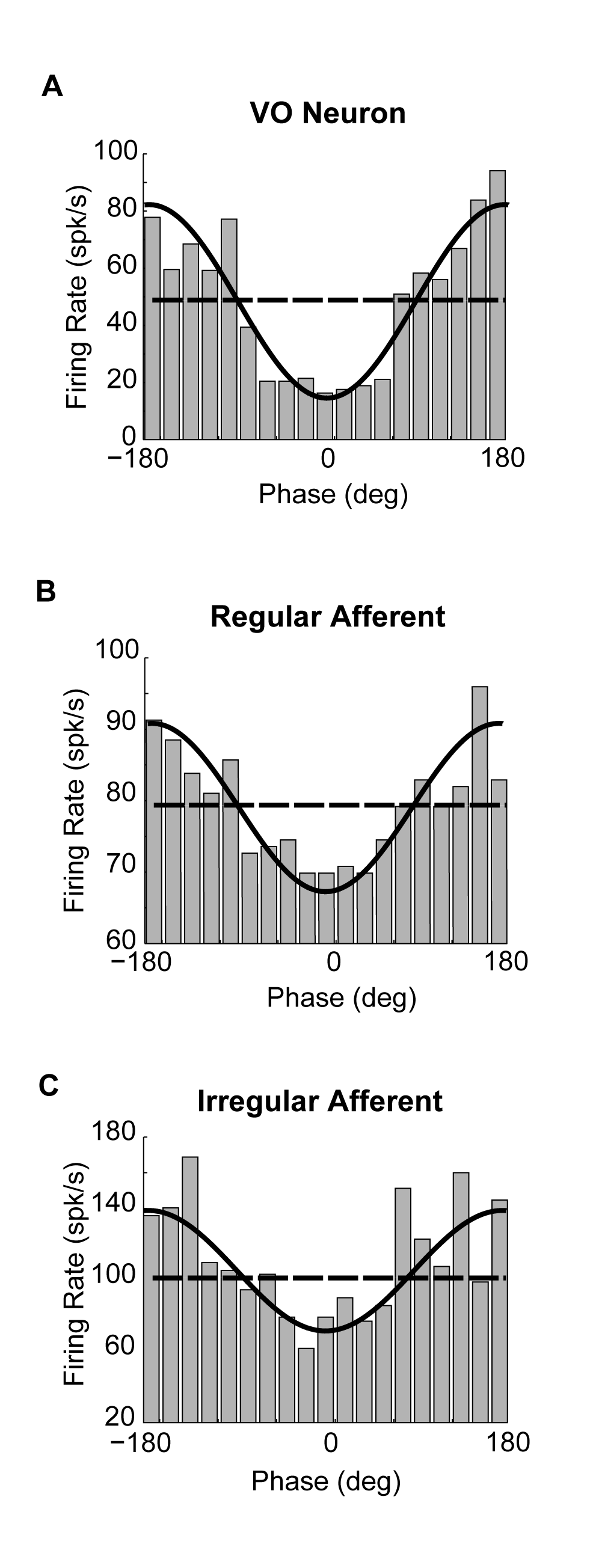

Supplement: Figure S4 — Central VO neurons as well as afferents do not show rectification and/or saturation when stimulated by the low and high frequency head rotations used in this study. (A–C) Phase histograms for an example VO neuron (A), regular afferent (B), and irregular afferent (C). The solid curves show the best sinusoidal fits. The dashed lines indicate the mean firing rates. Note that in no case do the histograms display either saturation or rectification. The population-averaged percentage of bins in the phase histograms corresponding to values less than 5% of the mean was 0 in more than 98% of cases, indicating no significant rectification. This was also true for 3 Hz and 3+17 Hz sinusoidal rotation (unpublished data) and for all neurons in the population. The population-averaged Variance-Accounted-For (VAF) of the sinusoidal fit for all three types of neurons was not significantly different between the different sinusoidal stimuli (p>0.15, t tests). This was also true for the noise stimuli (unpublished data). (TIF) [file pbio.1001365.s004.tif]

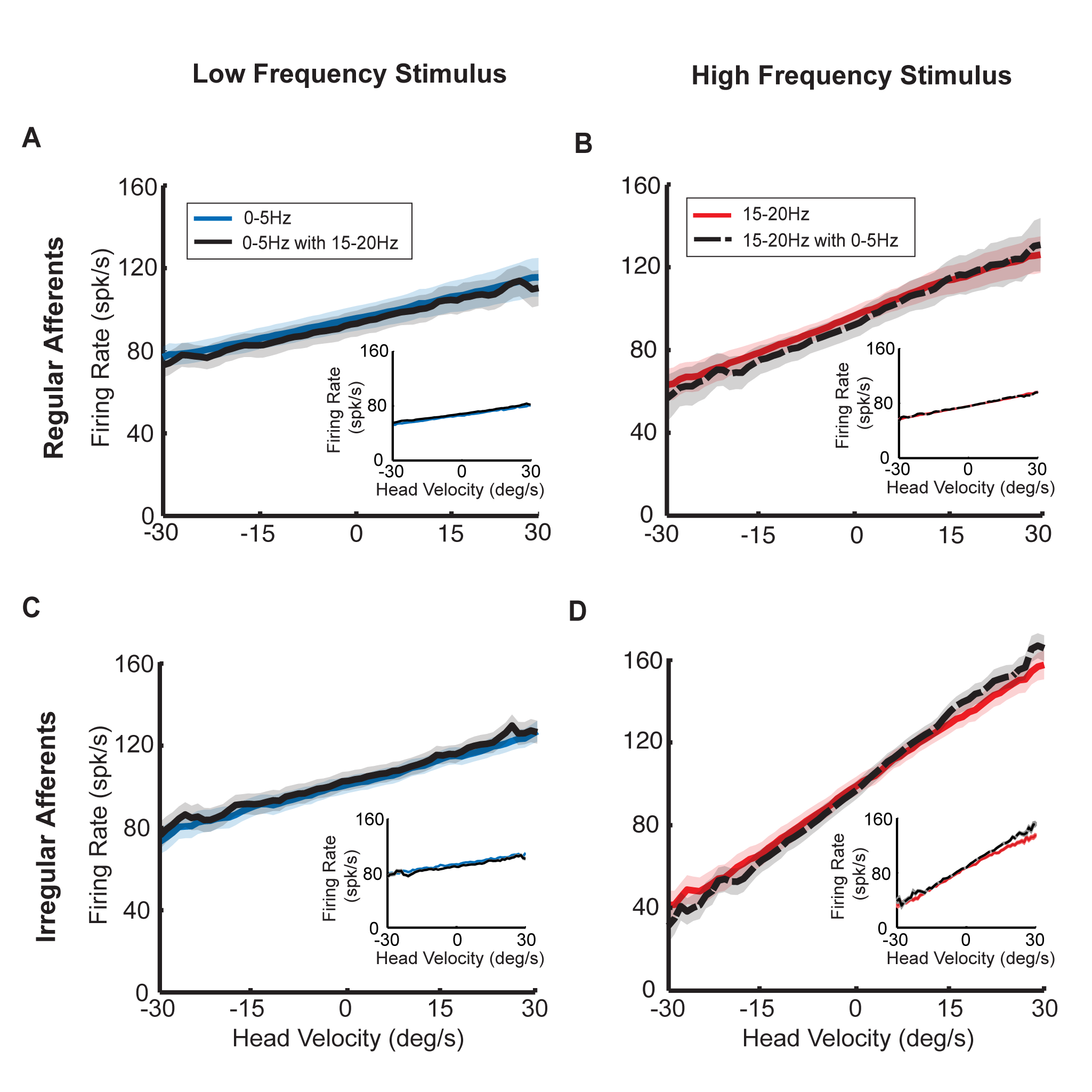

Supplement: Figure S5 — Afferents display a linear relationship between output firing rate and input head velocity. (A) Population-averaged firing rate as a function of head velocity for regular afferents when the low frequency (0–5 Hz) noise stimulus was applied in isolation (blue) and concurrently with the high frequency (15–20 Hz) noise stimulus (black). Inset: firing rate as a function of head velocity for an example regular afferent. (B) Population-averaged firing rate as a function of head velocity for regular afferents when the high-frequency (15–20 Hz) noise stimulus was applied in isolation (red) and concurrently with the low frequency (0–5 Hz) noise stimulus (dashed black). Inset: firing rate as a function of head velocity for the same regular afferent. (C) Population-averaged firing rate as a function of head velocity for irregular afferents when the low frequency (0–5 Hz) noise stimulus was applied in isolation (blue) and concurrently with the high frequency (15–20 Hz) noise stimulus (black). Inset: firing rate as a function of head velocity for an example irregular afferent. (C) Population-averaged firing rate as a function of head velocity for irregular afferents when the high-frequency (15–20 Hz) noise stimulus was applied in isolation (red) and concurrently with the low frequency (0–5 Hz) noise stimulus (dashed black). Inset: firing rate response as a function of head velocity for the same irregular afferent. (TIF) [file pbio.1001365.s005.tif]

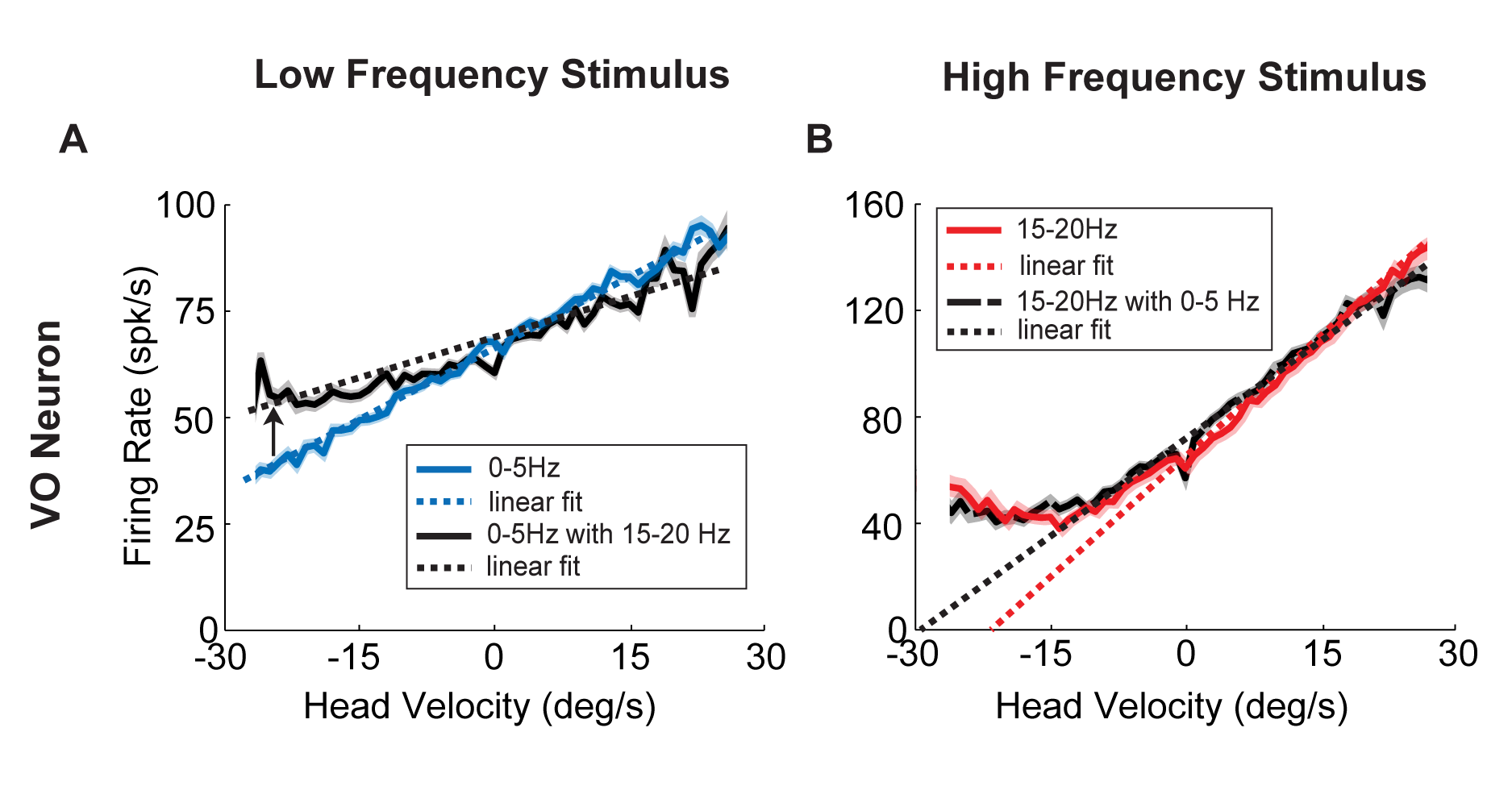

Supplement: Figure S6 — Individual central neurons display nonlinear responses. (A) Firing rate as a function of head velocity for an example central VO neuron when the low frequency (0–5 Hz) noise stimulus was applied in isolation (blue) and concurrently with the high frequency (15–20 Hz) noise stimulus (black). Both curves were well fit by straight lines (dashed lines). (B) Firing rate as a function of head velocity for the same example central VO neuron when the high frequency (15–20 Hz) noise stimulus was applied in isolation (red) and concurrently with the low frequency (0–5 Hz) noise stimulus (long dashed black). Note that both curves were not well fit by straight lines (short dashed lines). (TIF) [file pbio.1001365.s006.tif]

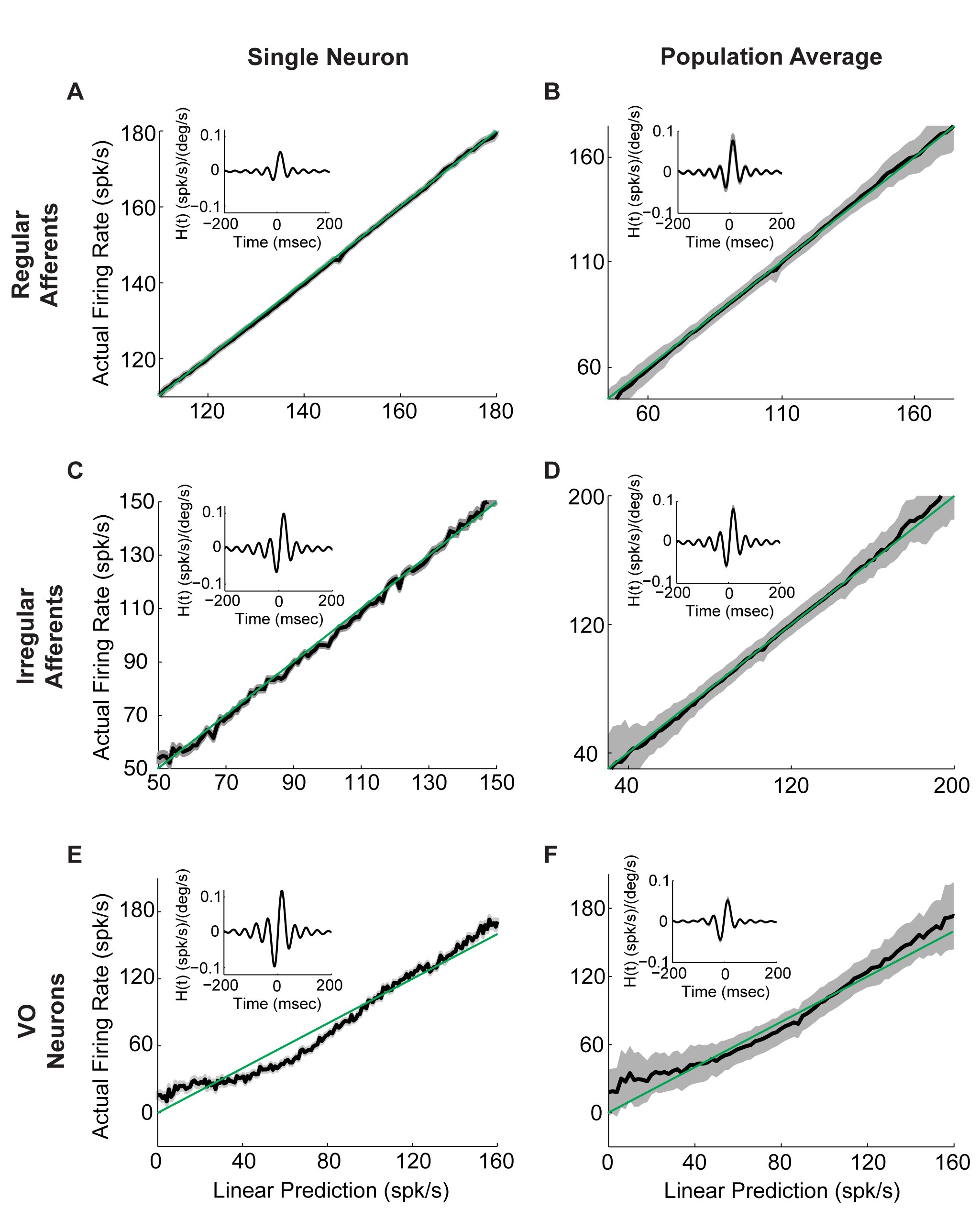

Supplement: Figure S7 — Characterization of central VO neurons and afferents by LN cascade models. (A) Actual firing rate as a function of the linear prediction for an example regular afferent. Inset: the filter H(t) for this same afferent. (B) Population-averaged actual firing rate as a function of the linear prediction for regular afferents. Inset: population-averaged filter H(t) for regular afferents. (C) Actual firing rate as a function of the linear prediction for an example irregular afferent. Inset: the filter H(t) for this same afferent. (D) Population-averaged actual firing rate as a function of the linear prediction for irregular afferents. Inset: population-averaged filter H(t) for irregular afferents. (E) Actual firing rate as a function of the linear prediction for an example central VO neuron. Inset: the filter H(t) for this same VO neuron. (F) Population-averaged actual firing rate as a function of the linear prediction for central VO neurons. Inset: population-averaged filter H(t) for central VO neurons. Throughout, the identity line is shown in green. (TIF) [file pbio.1001365.s007.tif]

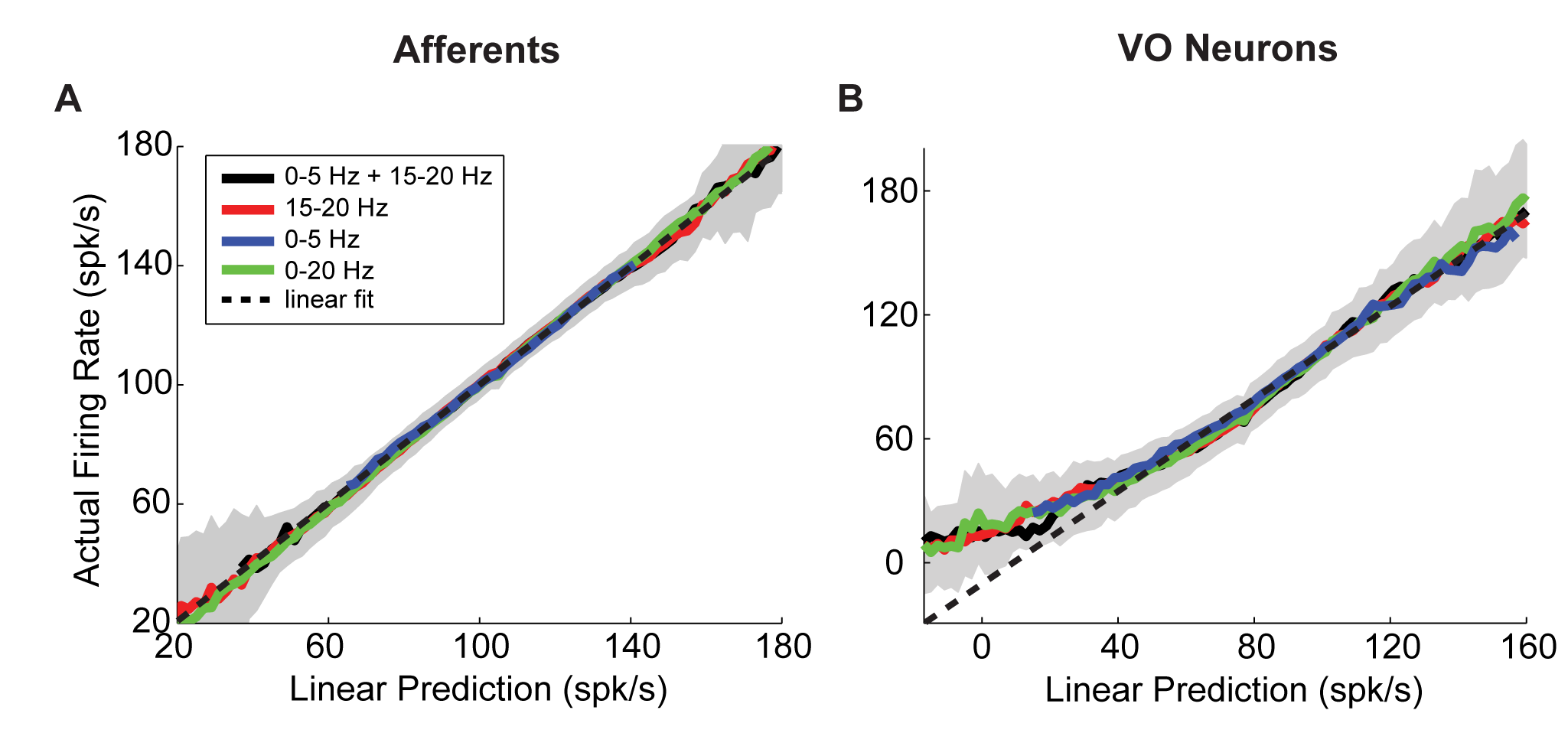

Supplement: Figure S8 — LN analysis reveals that central vestibular neurons but not afferents display a static nonlinearity in response to different self-motion stimuli. (A) Population-averaged actual firing rate as a function of the linear prediction for afferents in response to 0–20 Hz noise (green), 0–5 Hz noise (blue), 15–20 Hz noise (red), and 0–5 Hz+15–20 Hz noise (black). Note that all the curves are linear and overlap but that the blue curve extends over a narrower range than all the others. All the curves were further well fit by straight lines (R 2 = 0.99 in all cases). (B) Population-averaged actual firing rate as a function of the linear prediction for central VO neurons in response to 0–20 Hz noise (green), 0–5 Hz noise (blue), 15–20 Hz noise (red), and 0–5 Hz+15–20 Hz noise (black). Note that all the curves overlap but that the blue curve extends over a narrower range than all the others. As such, the blue curve is relatively better fit by a straight line (0–5 Hz: R 2 = 0.91; 15–20 Hz: R 2 = 0.58; 0–5 Hz+15–20 Hz: R 2 = 0.37; 0–20 Hz: R 2 = 0.62). (TIF) [file pbio.1001365.s008.tif]
